# Rotaviruses in Pigeons With Diarrhea: Recovery of Three Complete Pigeon Rotavirus A Genomes and the First Case of Pigeon Rotavirus G in Europe

**DOI:** 10.1155/tbed/4684235

**Published:** 2024-11-25

**Authors:** Ewa Łukaszuk, Daria Dziewulska, Tomasz Stenzel

**Affiliations:** Department of Poultry Diseases, Faculty of Veterinary Medicine, University of Warmia and Mazury in Olsztyn, Olsztyn, Poland

**Keywords:** complete genome, diarrhea, droplet digital PCR, Oxford Nanopore sequencing, pigeon, rotavirus

## Abstract

Rotaviruses are well-recognized pathogens responsible for diarrhea in humans and various animal species, with *Rotavirus A* the most often detected and most thoroughly described. Rotaviral disease is an important concern in pathology of pigeons as well, as pigeon rotavirus A was proven to play a major role in young pigeon disease (YPD). However, rotaviruses of other groups have been so far understudied in birds. This paper describes the first finding of Rotavirus G in domestic pigeon in Europe, as well as the recovery of three complete genomes of pigeon rotavirus A with Oxford Nanopore Sequencing. Quantification of pigeon rotavirus A genetic material with droplet digital polymerase chain reaction (PCR) in pigeons suffering from diarrhea and in asymptomatic pigeons was also performed in the frameworks of this study and resulted in determination of statistically highly significant differences between the groups in both detection rate and shedding of the virus. Phylogenetic analysis revealed the close relationship of acquired strains with those originating from pigeons from Europe, North America, Asia, and Australia, indicating a broad geographical spread of pigeon rotaviruses. Results of our research shed more light on occurrence and diversity of *Rotavirus* species in pigeons.

## 1. Introduction

Rotaviruses, namely, viruses of *Rotavirus* genus in *Sedoreoviridae* family, have icosahedral virions with surface spikes giving them a wheel-like appearance, which is the reason for the name of the genus (from Latin *rota*–wheel). Their capsid is composed of three protein layers that surround a genome organized in 11 segments of linear double-stranded RNA. The segments contain genes encoding six structural viral proteins (VPs) present in every viral particle and five or six nonstructural proteins (NSPs) produced only in rotavirus-infected cells [1, 2]. The genus currently consists of nine species: *Rotavirus A*, *B*, *C*, *D*, *F*, *G*, *H*, *I*, and *J* [3]. Viruses belonging to *Rotavirus A* (RVA) species have been the most thoroughly researched and described and a system allowing scientists to assign each segment to a genotype based on nucleotide identity has been established [4]. Group A rotaviruses are very commonly detected in various species of vertebrates and have also been found in multiple avian species, including chicken, turkey, guinea fowl, pheasant, domestic pigeon and in wild birds [5–12]. In turn, finding of *Rotavirus D*, *F*, and *G* (RVD, RVF, and RVG) has only been documented in birds and information on them are far more scarce [9, 13–20]. While not much is known about the pathogenicity of the latter, RVA is a well-known pathogen responsible for diseases of the gastrointestinal tract associated with diarrhea in both mammals and birds [11, 21]. Enteric diseases, especially those of viral origin, are a serious issue in medicine of racing pigeons as well. Health conditions affecting gastrointestinal tract cause weakened water and nutrient absorption, thus leading to dehydration, weight loss, and poor racing performance. Moreover, some of the viruses causing gastrointestinal illness require costly diagnostic tools to be detected, are not susceptible to routine therapeutic methods, and can provide suitable conditions for other pathogens to infect the vulnerable host. All of these factors contribute to high significance of viral infection in these birds.

Rotaviruses are prevalent in pigeons and cases of RVA in either clinically sick or asymptomatic pigeons have been reported in numerous countries around the world [5, 9, 10, 22–28]. RVA plays a major role in young pigeon disease (YPD) and pathogenicity of pigeon rotavirus A for pigeons has been proven by Rubbenstroth et al. [29]. Interestingly, a few reports on presence of rotaviruses of other groups have also been made. Complete genome sequence of RVG has been recovered from pigeon feces in China [17], and RVD genetic material has been detected with quantitative polymerase chain reaction (qPCR) in fecal samples collected from pigeons in Nigeria [9]. Unfortunately, these are the only findings of these viruses in pigeons available to date and because in both cases the health status of the birds was unknown, no connection between the presence of RVD and RVG and disease in pigeons can be observed.

This paper describes our efforts to find and quantify genetic material of pigeon rotavirus A in young racing pigeons, both clinically healthy and exhibiting signs of enteric disease such as diarrhea, but without the typical signs of YPD, like vomiting and congested crop. To do so, we utilized droplet digital PCR (ddPCR), which is a state-of-the art molecular biology method that allows to directly quantify the number of viral genome copies in the sample. The samples positive in this assay were subjected to third-generation sequencing to retrieve complete *Rotavirus* genome sequences.

## 2. Materials and Methods

### 2.1. Ethics Statement

The samples examined in this study were fecal samples and cloacal swabs, the collection of which did not require an ethical approval. The fecal samples were provided by the pigeon owners, and cloacal swabs were collected during the standard clinical examination, to which the owners consented.

### 2.2. Sample Collection

Selection of pigeons for the study and collection of the samples was performed in Poland in 2023, as described in our previous studies [30, 31]. Swab samples were collected from 90 pigeons with signs of enteric disease, originating from 24 flocks, and from 63 asymptomatic pigeons, originating from 13 flocks. Sick and asymptomatic birds were considered the study group (S) and the control group (C), respectively. Pigeons qualifying for the group S had presented clinical signs for no longer than a week, and pigeons qualifying for the group C originated from flocks without current or recent disease outbreaks. After collection, the swabs were suspended in the UTM Universal Transport Medium (Copan Diagnostics, Murrieta, California, USA), and the medium of every sample was divided into two parts and then stored at −80°C.

### 2.3. Extraction of RNA

RNA was extracted from the first part of the transport medium of each sample using Total RNA Mini Plus kit (A&A Biotechnology, Gdańsk, Poland) and eluted in 50 μL of nuclease-free water. Then a NanoDrop 2000 Spectrophotometer (Thermo Fisher Scientific, Waltham, Massachusetts, USA) was used to measure purity and concentration of the samples, and the samples were then stored at −80°C until testing.

### 2.4. Quantitative Analysis

#### 2.4.1. TaqMan qPCR

As the first part of quantitative analysis, TaqMan qPCR was performed as an assay necessary to assess Cq values. Primers and probes from the method developed by Rubbenstroth et al. [24] were used in TaqMan qPCR to test the samples for the presence of pigeon rotavirus A genetic material. The reaction mix consisted of 10 µl of TaqMan Fast Universal PCR Master Mix kit (Thermo Fisher Scientific, Waltham, Massachusetts), 0.6 µL of forward primer, 1.8 µL of reverse primer, 2 µL of probe, 2.6 µL of RNase-free water, and 3 µL of cDNA. Positive control for the assay consisted of a sample originating from the collection of Department of Poultry Diseases, Faculty of Veterinary Medicine, University of Warmia and Mazury in Olsztyn, Poland. The conditions of the reaction carried out in the LightCycler 96 System thermocycler (Roche, Basel, Switzerland) were as follows: 95°C for 30 s and 40 cycles of 95°C for 15 s and 60°C for 40 s. All samples were analyzed in duplicate and those with Cq of 35 or less were considered positive. To ensure proper readability of ddPCR results, samples with Cq of 25 or lower were diluted with nuclease-free water, assuming that one tenfold dilution increases Cq by 3.

#### 2.4.2. ddPCR

As a second and final step of quantitative analysis, samples testing positive in TaqMan qPCR were subjected to ddPCR. First, 22 μL of reaction mixture was prepared by mixing 11 µL of ddPCR Supermix for Probes (Bio-Rad, Hercules, California, USA), 1.98 μL of primers and 2.2 µL of probe same as in TaqMan qPCR, 1.84 µL of RNase-free water, and 3 µL cDNA. Then, the droplet emulsions were obtained as described in our previous studies [30, 31]. The positive control for the ddPCR reaction was the same as in the TaqMan qPCR, and all samples were analyzed in duplicate. The conditions of the ddPCR reaction conducted in C1000 Touch Thermal Cycler (Bio-Rad, Hercules, California, USA) were as follows: 95°C for 10 min, 40 cycles of 95°C for 30 s and 50°C for 1 min, and 4°C for 30 min. The numbers of viral amplicons in the droplets were calculated with QX 200 Droplet Reader (Bio-Rad, Hercules, California, USA). In case of samples diluted prior to ddPCR, the values were multiplied by the dilution value. The final results were presented as mean number of copies of pigeon rotavirus A amplicons ± standard deviation per 20 µL of the reaction mixture.

### 2.5. Oxford Nanopore Sequencing

The next part of the study was third-generation sequencing outsourced to PathoSense laboratory (Oxford Nanopore Technologies Certified Service Provider, Merelbeke, Belgium). The remaining part of the transport medium of all samples from both investigated groups positive for pigeon rotavirus A genetic material in TaqMan qPCR was purified with 0.8 µm polyethersulphone spin filters (Sartorius, Goettingen, Germany) to enrich for viral particles. Then, nuclease treatment was performed, nucleic acids were extracted, the samples were enriched for DNA and RNA, and the libraries were prepared as previously described [32–34]. GridION X5 sequencer (Oxford Nanopore Technologies Ltd., Oxford, United Kingdom) with a R9.4.1 flow cell in combination with Rapid Barcoding Kit SQK-RBK110-96 (Oxford Nanopore Technologies Ltd., Oxford, United Kingdom) used for library preparation were employed to perform sequencing for 24 h. In-house bioinformatic pipelines were used to convert the acquired raw data to bases with Guppy v7.1.4, quality filter, and taxonomically classify. Finally, Canu v2.2 [35] and Medaka v1.4.1 (Oxford Nanopore Technologies Ltd., Oxford, United Kingdom) tools were used to de novo assemble the viral genome sequences.

### 2.6. Bioinformatic Analysis

The Basic Local Alignment Search Tool (BLAST) search [36] using megablast and discontignous megablast algorithms was performed on sequences acquired with Oxford Nanopore sequencing to find similar sequences of rotaviruses available in GenBank database. In case of lack of hits, the search was re-run with the blastn algorithm. This way, a separate set of sequences was prepared for every genome segment, the sequences were aligned with MAFFT method [37], open reading frames (ORFs), and complete coding sequences (CDs) were determined with Find ORFs tool, and the CDs were translated to amino acids with Translate tool, all in Geneious Prime v. 2024.0.3 software (Dotmatics, Boston, Massachusetts, USA). Each segment of RVA strains was assigned to genotype based on classification system proposed by Matthijnssens et al. [4]. Then, substitution models most accurate for phylogenetic analysis were established with Find DNA/protein models tool in MEGA 11 software [38], and maximum likelihood analysis with 1000 bootstrap replicates were performed for each genome segment in IQ-TREE 1.6.12 software [39, 40]. The resulting phylogenetic trees were visualized with iTOL v6 software [41].

In addition, because members of species within the *Rotavirus* genus are often identified by amino acid identity of the VP6 no lower than 53% [42], a set of complete VP6 sequences of RVA, RVB, RVC, RVD, RVF, RVG, RVH, RVI, and RVJ available in GenBank along with complete VP6 sequences obtained in this study was prepared to demonstrate the diversity among the *Rotavirus* species. The amino acid identities were calculated, and pairwise identity matrix was created in SDT v1.3 software [43] Finally, to acquire accession numbers, all the obtained sequences were deposited in the National Center for Biotechnology Information (NCBI) GenBank database, and the metagenomic data were deposited in the NCBI BioProject database.

### 2.7. Statistical Analysis

The correlation of the detection rate of pigeon rotavirus A with the health status of the pigeons was assessed with the chi-square test (*χ*^2^). In turn, the correlation of the amount of the genetic material of pigeon rotavirus A in positive samples with the health status of the pigeons was assessed with the nonparametric Mann–Whitney *U* test. Differences were considered significant with *p* < 0.05. Statistical analysis was made with Statistica 13 software (Statsoft, Cracow, Poland).

## 3. Results

### 3.1. Quantitative Analysis

#### 3.1.1. TaqMan qPCR

**Figure 1:**
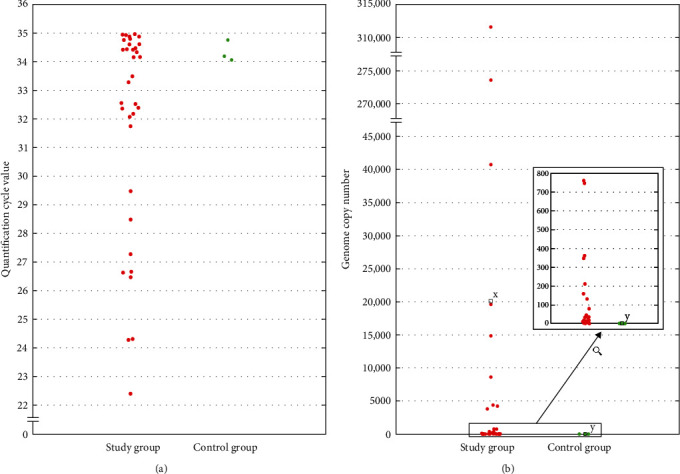
(a) Results of TaqMan qPCR assay for pigeon rotavirus A genetic material. Quantification cycle (Cq) values are presented for samples from the study and control group. (b) Results of absolute quantification of pigeon rotavirus A genetic material with ddPCR method in positive samples from the study and control group, expressed as mean genome copy number per 20 μL of the reaction mixture. Blank squares represent mean values in both groups. Letters *x* and *y* indicate the statistically highly significant difference in genome copy number between the two groups (*p*=0.0050).

TaqMan qPCR showed that 34 samples out of 90 (37.8%) in group S and three samples out of 63 (4.8%) in group C were positive for pigeon rotavirus A genetic material and the difference between the groups was found highly significant in the chi-square test (*χ*^2^=22.03, *p*=0.001). The positive samples were taken from birds originating from 12 and 3 flocks from the groups S and C, respectively. There were three flocks from the group S in which all the sampled birds were found positive and every positive bird from group C originated from a different flock. The results of qPCR are visualized in Figure 1a.

#### 3.1.2. ddPCR

The absolute quantification of pigeon rotavirus A genetic material with ddPCR revealed the mean genome copy number in 20 µL of the reaction mixture as 20,136.34 ± 69,753.68 and 1.53 ± 0.12 for the S and C groups, respectively. The difference between the groups was found highly significant in the Mann–Whitney *U* test with *p*=0.005. The results of ddPCR are visualized in Figure 1b.

### 3.2. Bioinformatic Analysis

#### 3.2.1. Pigeon Rotavirus Genome Recovery

**Figure 2:**
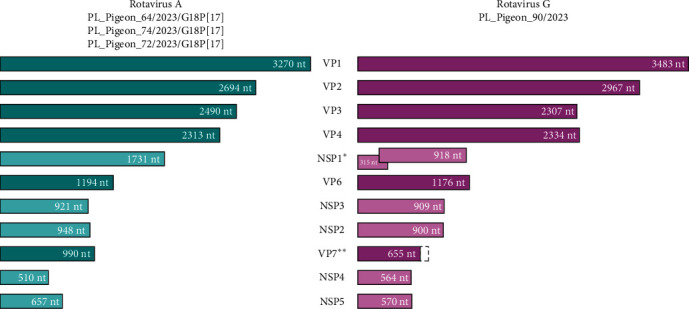
Schematic illustration of the genome segments of pigeon rotavirus A and pigeon rotavirus G sequences obtained in this study. Genome segments encoding structural proteins and nonstructural proteins are colored in darker and brighter shades of the colors, respectively. *⁣*^*∗*^NSP1-1 and NSP1-2 in case of pigeon rotavirus G strain. *⁣*^*∗∗*^VP7 sequence of pigeon rotavirus G strain is incomplete, consisting of ~88% length of reference pigeon sequences.

Forty-four genome segments, forming genomic sequences of four distinct strains, were obtained by third-generation sequencing. Three strains were found to belong to RVA species and one to RVG species. Complete genome sequences were obtained for RVA strains, while the sequence determined for RVG strain PL_Pigeon_90/2023 was missing 89 nucleotides on 3′ end of VP7 segment. All RVA strains were found to belong to the genotype G18-P [17]-I4-R4-C4-M4-A4-N4-T4-E19-H4. The nucleotide sequences of individual genome segments of each strain received GenBank accession numbers PP849416-PP8494156, PQ468958-PQ468959, and PQ479264, while all metagenomic data can be found in the BioProject database with accession number PRJNA1114660 under BioSample accession numbers SAMN41490570-SAMN41490573. Information on the sequences obtained in this study is summarized in Table 1, and the organization of the segments forming the RVA and RVG genomes is presented in Figure 2.

All strains were acquired from pigeons originating from four different flocks of group S. RVA strains were detected in pigeons suffering from diarrhea and apathy for 3–5 days prior sampling. In turn, RVG was detected in a pigeon originating from flock exhibiting diarrhea, lack of appetite, and apathy for 3 days prior sampling.

#### 3.2.2. Genome Organization

Genomes of all obtained strains were of similar structure to other representants of the respective species. ORF of all sequences started with the AUG codon. Average pairwise identities of the sequences obtained in this study and of other pigeon sequences are presented in *Supporting Information*1. Analysis of the nucleotide and amino acid identities for segments VP4 and NSP4 of RVG revealed an exception among other segments—the pigeon RVG strain obtained in this study shared the highest pairwise identities with RVG strains acquired from chickens (accession numbers PP228909 and KJ752082), measuring 64.10 and 58.50% of nucleotide and amino acid identity, respectively, in case of VP4; and 81.90 and 79.70% of nucleotide and amino acid identity, respectively, in case of NSP4. In all the other cases, strains acquired from pigeons shared the highest identity scores.

**Figure 3:**
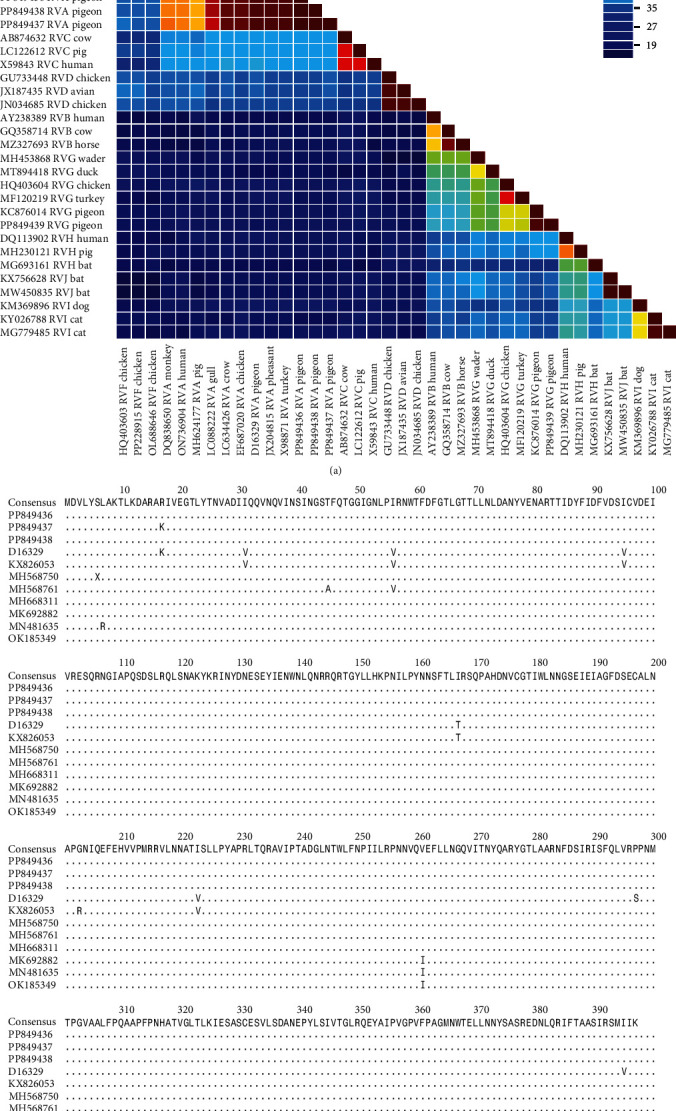
Pairwise identity matrix of the VP6 amino acid sequences of rotaviruses obtained in this study and representants of all rotavirus species (*Rotavirus A*, *B*, *C*, *D*, *F*, *G*, *H*, *I*, and *J*) available in the GenBank database. All sequences are labeled with the accession number, abbreviation of the virus name, and host name. The labels of the sequences obtained in this study are written in bold (a). Alignment of VP6 amino acid sequences of pigeon rotavirus A strains obtained in this study and available in the GenBank database. Dots indicate amino acids consistent with the consensus (b).

Figure 3a shows the pairwise identity matrix of the complete VP6 amino acid sequences of rotaviruses obtained in this study and other representant rotaviruses available in the GenBank database. The average pairwise identity was 87.08 and 65.07% between the analyzed VP6 amino acid sequences of *Rotavirus A* and *G* strains, respectively. Figure 3b shows the alignment of VP6 amino acid sequences of pigeon rotavirus A strains.

#### 3.2.3. Phylogenetic Analysis

**Figure 4:**
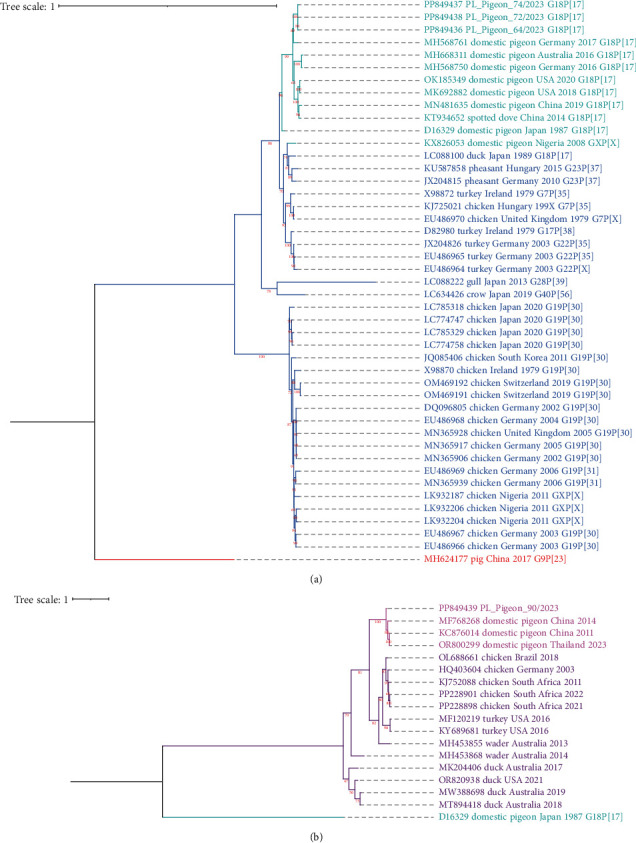
Phylogenetic trees of nucleotide sequences of *Rotavirus* genome segments encoding VP6. The trees consist of *Rotavirus A* (a) and *Rotavirus G* (b) sequences obtained in this study and representant complete sequences acquired from GenBank database. RVA tree is rooted with the VP6 sequence of pig rotavirus A isolate RVA/Pig/China/SC11/2017/G9P [23] while RVG tree is rooted with the VP6 sequence of avian rotavirus PO-13. The trees were inferred in IQ-TREE 1.6.12 software [39, 40] and visualized with iTOL v6 software [41]. The distances were calculated with the maximum likelihood method with 1000 bootstrap replicates. The substitution model used was TN93+G as it was found most appropriate for both alignments with Find DNA/protein models tool in MEGA 11 software [38].

Figure 4 depicts phylogenetic trees created of complete nucleotide sequences of VP6 segment of RVA (a) and RVG (b). To infer the trees, we used TN93 + G substitution model. In turn, the trees composed of complete nucleotide sequences of all the other segments of RVA and RVG can be found in the *Supporting Information*2 and 3, respectively. In all trees, both pigeon RVA and pigeon RVG sequences formed separate clades, showing the close genetic relationship of sequences of individual *Rotavirus* species derived from pigeons. As the inferred trees show, all pigeon RVA sequences obtained in this study clustered together with strains originating from Germany, as well as North American, Chinese, and Australian strains. Based on analysis of all genomic segments, our strains were found to be most closely related to those recovered in Germany and Australia between 2012 and 2018. As for RVG, the sequences of all genome segments recovered in this study clustered together with pigeon strains originating from China and Thailand. It can also be noted that in case of trees depicting the relationship of segment 4 sequences encoding VP4 and of segment 10 sequences encoding NSP4, pigeon RVG strain obtained in this study appears to be more closely related to strains originating from chickens than to other pigeon strains.

## 4. Discussion

Rotaviral infections pose an important health problem in racing pigeons, inducing signs of enteric disease that compromise their racing performance. Similarly to other viral infections, the determination of the agent responsible for the disease and assessment of prognosis can be difficult because of the possibility of numerous factors contributing to the manifestation of clinical signs. Quantitative methods could be useful in evaluating the severity of viral infection, as a high number of viral amplicon copies in the clinical sample might indicate an ongoing disease and virus multiplication [44]. To date, no method of pigeon rotavirus A quantification has been described other than a semiquantitative method based on *Cq* value [24, 28]. In our study, we decided to use an assay that was recently implemented in our laboratory and successfully used to quantify other viruses—ddPCR [30, 31, 44–46]. The main advantage of this method is that the tolerance to PCR inhibitors is enhanced compared to qPCR. This way it can help to evaluate viral infection in inhibition-prone samples such as stool, sputum, and tissue samples [47–49].

TaqMan qPCR revealed a high detection rate of pigeon rotavirus A in the group of clinically ill pigeons, remarkably higher than in the asymptomatic control group, where detection of the genetic material of the virus appears to be incidental, especially considering that each positive case in group C was a single finding in an individual flock. It is also impossible to overlook the difference in viral shedding assessed with ddPCR, which was over 13,000 times higher in group S comparing to group C. Considering that the differences between the two groups in both applied statistical tests turned out to be highly significant, it can be assumed that pigeon rotavirus A was indeed the reason of illness in the tested pigeons. It is in accordance with the information available in literature on this topic—pigeon rotavirus A is one of the few viruses found in pigeons, for which the pathogenicity was proved with fulfilling the Henle-Koch postulates [29]. However, it should be noted that the typical clinical presentation of rotaviral infection in pigeons differs from that observed in pigeons sampled in our study—the described clinical cases connected with RVA detection usually manifested with lack of appetite, regurgitation, congested crop, diarrhea, emaciation and in some cases death, and hepatic necrosis could be found in histopatology [10, 23–25]; while the most pigeons of group S presented only with diarrhea and apathy and no deaths were observed in most of the flocks of origin. Because our research did not involve euthanizing the pigeons, unfortunately, it is impossible to compare the anatomopathological and histopathological findings. On the one hand, one may take into consideration that in the most of the aforementioned cases other pathogens like pigeon circovirus, bacteria, and intestinal parasites might have been the disease triggers [50–52]. On the other hand, Rubbenstroth et al. [29] proved that sole infection of young pigeons with pigeon rotavirus A can result in a disease presenting similar to YPD. However, in case of our study, while bacterial, fungal, or parasitical infection was excluded as a cause of disease, it is possible that the presence of other viruses affected the clinical symptoms. Further studies would be beneficial in assessing how infection with multiple viruses might impact the course of gastrointestinal disease in pigeons. The final thing worth considering here is that all pigeons of group S sampled in this study showed clinical symptoms for no longer than a week, and thus at the peak point of the disease, so that the form of rotaviral infection described in this paper can be considered mild compared to this reported by the other researchers. More research on this topic would be required to assess if the strains acquired in this study are less pathogenic in comparison to those obtained by Rubbenstroth et al. [24], McCowan et al. [10] and others.

Third-generation sequencing also resulted in undoubtedly interesting findings. The three recovered RVA strains were found to belong to genotype G18-P [17]-I4-R4-C4-M4-A4-N4-T4-E19-H4, which is the same as pigeon rotavirus A detected in pigeons from North America, Germany, and Australia [10, 24, 27]. This suggests the wide geographical spread of these viruses. Pigeon racing and trade might be the reason for that—pigeon sport is an activity of undiminished popularity, and birds that often travel hundreds of kilometers and are often susceptible to infections because of the tremendous physical effort incurred can be vectors of various pathogens [53, 54]. Because pigeon flying freely may stop to rest on livestock buildings, infectious agents can be potentially transmitted not only between individuals of the same species but also between other species. The evidence of this might be, for example, the finding of the genetic material of avian rotavirus PO-13, originating from a pigeon, in feces of a calf with diarrhea in Germany [55]. This is why it is so important to explore pigeon diseases and develop methods of prevention and control.

The obtained RVA strains were found to be highly similar to each other and to the other known pigeon RVAs, with differences even as small as single amino acids in the complete VP6 amino acid sequence, which is thought to be the most conserved. The amino acid identities for the other segments vary between 91.07 and 98.43% on average. All these findings further support our theory of broad geographical spread of the virus.

Although our initial intention for this study was to screen the samples for the presence of RVA genetic material and to further investigate genetic diversity of polish RVA strains with third-generation sequencing, metagenomics allowed us to obtain a sequence of rotavirus of another group. This paper describes the first detection of RVG in pigeons outside Asia which is a rather remarkable discovery. We were able to recover complete sequences of 10 out of 11 genomic segments and a partial sequence of a single segment of pigeon rotavirus G. Group G rotaviruses have noticeably greater genetic diversity than RVAs, which is also reflected in our results. The nucleotide and amino acid identities between the obtained RVG sequences and other pigeon RVG sequences were lower than in case of RVA for all genomic segments. VP6 was found to be the most conserved gene with average 98.67% amino acid identity, which corresponds to current knowledge [15, 41, 56]. What is interesting, the gene with the lowest nucleotide and amino acid identity was VP4, where NSP1 is considered to be the most variable part of the genome [2]. However, this assumption is probably based on the well-researched rotaviruses of group A and may not be directly applicable to rotaviruses of group G which seem to be much more diverse. It is also worth mentioning that the RVG strain with the highest amino acid identity in VP4 to pigeon RVG obtained in this study was acquired from a chicken from South Africa. The phylogenetic tree of VP4 gene sequences also shows the close relationship of the novel pigeon RVG strain with those found in chickens, suggesting a possible common origin of this genome segment in distant bird species and further supporting our insight on broad geographic spread of avian rotaviruses (*Supporting Information*3d). The thorough analysis and comparison of RVG are hindered by a low number of sequences available in the GenBank database and even lower number of publications on this subject, especially in case of pigeon rotavirus G. In this study, RVG was found in the sample acquired from a diseased pigeon; however, there is no evidence on potential pathogenicity of this virus, as the only described detection involved feces of pigeons of unknown health status [17], and unfortunately we are unable to draw any conclusions because (i) quantitative analysis of pigeon rotavirus G was not performed in the framework of this study and (ii) only one pigeon was positive for genetic material of this virus.

## 5. Conclusions

Our research describes detection of complete genomes of three RVA strains. One RVG strain has also been detected, which is the first case of RVG infection in a pigeon in Europe. These findings prove broad geographic distribution of these important pathogens. We were also able to reveal statistically highly significant differences between the diarrheic and asymptomatic group in both detection rate and shedding of the virus, using quantitative methods. We hope that the obtained results broaden the knowledge on diversity of rotaviruses in pigeons.

## Figures and Tables

**Table 1:** Summary of the coding sequences of all segments of the *Rotavirus* strains obtained in this study.

Genome segment	Protein	Species	Strain	Accession number	Length (nt)	GC (%)
1	VP1	RVA	PL_Pigeon_64/2023/G18P [17]	PP849424	3270	31.9
			PL_Pigeon_74/2023/G18P [17]	PP849425	3270	31.7
			PL_Pigeon_72/2023/G18P [17]	PP849426	3270	31.7
		RVG	PL_Pigeon_90/2023	PP849416	3483	34.5

2	VP2	RVA	PL_Pigeon_64/2023/G18P [17]	PP849427	2694	37.9
			PL_Pigeon_74/2023/G18P [17]	PP849428	2694	38.0
			PL_Pigeon_72/2023/G18P [17]	PP849429	2694	38.0
		RVG	PL_Pigeon_90/2023	PP849430	2967	35.7

3	VP3	RVA	PL_Pigeon_64/2023/G18P [17]	PP849431	2490	31.9
			PL_Pigeon_74/2023/G18P [17]	PP849417	2490	31.8
			PL_Pigeon_72/2023/G18P [17]	PP849432	2490	31.8
		RVG	PL_Pigeon_90/2023	PP849433	2307	33.5

4	VP4	RVA	PL_Pigeon_64/2023/G18P [17]	PP849434	2313	35.0
			PL_Pigeon_74/2023/G18P [17]	PP849435	2313	34.9
			PL_Pigeon_72/2023/G18P [17]	PP849418	2313	34.8
		RVG	PL_Pigeon_90/2023	PQ468959	2334	35.0

5	NSP1*⁣*^*∗*^	RVA	PL_Pigeon_64/2023/G18P [17]	PP849419	1731	35.0
			PL_Pigeon_74/2023/G18P [17]	PP849420	1731	35.5
			PL_Pigeon_72/2023/G18P [17]	PP849421	1731	35.2
		RVG	PL_Pigeon_90/2023	PP849422	315 918	38.4 33.3

6	VP6	RVA	PL_Pigeon_64/2023/G18P [17]	PP849436	1194	39.2
			PL_Pigeon_74/2023/G18P [17]	PP849437	1194	39.1
			PL_Pigeon_72/2023/G18P [17]	PP849438	1194	39.1
		RVG	PL_Pigeon_90/2023	PP849439	1176	37.4

7	NSP3	RVA	PL_Pigeon_64/2023/G18P [17]	PP849440	921	37.7
			PL_Pigeon_74/2023/G18P [17]	PP849441	921	37.9
			PL_Pigeon_72/2023/G18P [17]	PQ468958	921	37.5
		RVG	PL_Pigeon_90/2023	PP849442	909	36.4

8	NSP2	RVA	PL_Pigeon_64/2023/G18P [17]	PP849443	948	35.0
			PL_Pigeon_74/2023/G18P [17]	PP849444	948	34.8
			PL_Pigeon_72/2023/G18P [17]	PP849445	948	35.0
		RVG	PL_Pigeon_90/2023	PP849423	900	38.9

9	VP7	RVA	PL_Pigeon_64/2023/G18P [17]	PP849446	990	36.0
			PL_Pigeon_74/2023/G18P [17]	PP849447	990	36.0
			PL_Pigeon_72/2023/G18P [17]	PP849448	990	36.1
		RVG	PL_Pigeon_90/2023	PQ479264	655*⁣*^*∗∗*^	34.2

10	NSP4	RVA	PL_Pigeon_64/2023/G18P [17]	PP849449	510	35.3
			PL_Pigeon_74/2023/G18P [17]	PP849450	510	35.7
			PL_Pigeon_72/2023/G18P [17]	PP849451	510	35.3
		RVG	PL_Pigeon_90/2023	PP849452	564	32.8

11	NSP5	RVA	PL_Pigeon_64/2023/G18P [17]	PP849453	657	33.8
			PL_Pigeon_74/2023/G18P [17]	PP849454	657	34.1
			PL_Pigeon_72/2023/G18P [17]	PP849455	657	33.8
		RVG	PL_Pigeon_90/2023	PP849456	570	31.2

*Note:* RVA and RVG are abbreviations for *Rotavirus A* and *Rotavirus G*, respectively.

*⁣* ^*∗*^NSP1-1 and NSP1-2 in case of RVG strain.

*⁣* ^*∗∗*^Partial sequence consisting of ~88% length of reference pigeon sequences, missing 89 nucleotides on 3′ end.

## Data Availability

The data that support the findings of this study are available from the corresponding author upon reasonable request. The nucleotide sequences obtained in this study were deposited in GenBank database under accession numbers PP849416–PP8494156, PQ468958–PQ468959, and PQ479264. The metagenomic data were deposited in the BioProject database with accession number PRJNA1114660 under BioSample accession numbers SAMN41490570–SAMN41490573.

## Supporting Information

Supporting Information 1 Average pairwise identities of the sequences obtained in this study and of other pigeon sequences acquired from GenBank database. SDT v1.3 software [43] was used to calculate the pairwise identities. *⁣*^*∗*^NSP1-1 and NSP1-2 in case of RVG strain.

Supporting Information 2 Phylogenetic trees of nucleotide sequences of Rotavirus A genome segments: VP1 (a), VP2 (b), VP3 (c), VP4 (d), NSP1 (e), NSP3 (f), NSP2 (g), VP7 (h), NSP4 (i), and NSP5 (j). The trees consist of sequences obtained in this study and complete avian RVA sequences acquired from GenBank database. All sequences are labeled with the accession number and host name, country and year of collection as well as simplified genotype designation, while the sequences obtained in this study are labeled with the accession number and strain name and written in bold. Each tree is rooted with the sequence of the corresponding genome segment of pig rotavirus A isolate RVA/Pig/China/SC11/2017/G9P [23]. The trees were inferred in IQ-TREE 1.6.12 software [39, 40] and visualized with iTOL v6 software [41]. The distances were calculated with the maximum likelihood method with 1,000 bootstrap replicates. The substitution models most appropriate for each alignment were calculated with Find DNA/protein models tool in MEGA 11 software [38] and are as follows: GTR+G+I for VP1, VP2, VP3, and VP4 sequences, GTR+G for NSP1, NSP3, NSP2, and VP7 sequences and HKY+G for NSP4 and NSP5 sequences.

Supporting Information 3 Phylogenetic trees of nucleotide sequences of Rotavirus G genome segments: VP1 (a), VP2 (b), VP3 (c), VP4 (d), NSP1-1 and NSP1-2 (e), NSP3 (f), NSP2 (g), VP7 (h), NSP4 (i), and NSP5 (j). The trees consist of sequences obtained in this study and complete RVG sequences acquired from GenBank database. All sequences are labeled with the accession number and host name as well as country and year of collection, while the sequences obtained in this study are labeled with the accession number and strain name and written in bold. Each tree is rooted with the sequence of the corresponding genome segment of avian rotavirus PO-13. The trees were inferred in IQ-TREE 1.6.12 software [39, 40] and visualized with iTOL v6 software [41]. The distances were calculated with the maximum likelihood method with 1000 bootstrap replicates. The substitution models most appropriate for each alignment were calculated with find DNA/protein models tool in MEGA 11 software [38] and are as follows: GTR+G for VP1, VP2, VP3, VP4, and VP7 sequences, TN93+G for NSP1-1 and NSP1-2, NSP3, NSP2, and NSP5 sequences and HKY+G for NSP4 sequences.
