# Supplementary material for: Rotaviruses in Pigeons With Diarrhea: Recovery of Three Complete Pigeon Rotavirus A Genomes and the First Case of Pigeon Rotavirus G in Europe
Source: Transbound Emerg Dis. 2024 Nov 25;2024:4684235. doi: 10.1155/tbed/4684235 (PMC12019971; doi:10.1155/tbed/4684235)
Supplement: Supporting Information 1 — Average pairwise identities of the sequences obtained in this study and of other pigeon sequences acquired from GenBank database. SDT v1.3 software [43] was used to calculate the pairwise identities. ⁣∗NSP1-1 and NSP1-2 in case of RVG strain. [file 4684235.f1.pdf]

<table>
<tr><th rowspan="3">Genome segment</th><th rowspan="3">Protein</th><th rowspan="3">Species</th><th colspan="8">Average pairwise identity [%]</th></tr>
<tr><th colspan="4">Between the obtained sequences</th><th colspan="4">Between the obtained sequences and other pigeon sequences</th></tr>
<tr><th colspan="2">Nucleotide identity</th><th colspan="2">Amino acid identity</th><th colspan="2">Nucleotide identity</th><th colspan="2">Amino acid identity</th></tr>
<tr><td rowspan="2">1</td><td rowspan="2">VP1</td><td>RVA</td><td colspan="2">99.43</td><td colspan="2">99.60</td><td colspan="2">94.81</td><td colspan="2">98.43</td></tr>
<tr><td>RVG</td><td colspan="2">-</td><td colspan="2">-</td><td colspan="2">88.43</td><td colspan="2">95.37</td></tr>
<tr><td rowspan="2">2</td><td rowspan="2">VP2</td><td>RVA</td><td colspan="2">99.60</td><td colspan="2">99.80</td><td colspan="2">93.72</td><td colspan="2">98.24</td></tr>
<tr><td>RVG</td><td colspan="2">-</td><td colspan="2">-</td><td colspan="2">86.40</td><td colspan="2">94.70</td></tr>
<tr><td rowspan="2">3</td><td rowspan="2">VP3</td><td>RVA</td><td colspan="2">99.60</td><td colspan="2">99.43</td><td colspan="2">93.95</td><td colspan="2">96.71</td></tr>
<tr><td>RVG</td><td colspan="2">-</td><td colspan="2">-</td><td colspan="2">85.17</td><td colspan="2">89.37</td></tr>
<tr><td rowspan="2">4</td><td rowspan="2">VP4</td><td>RVA</td><td colspan="2">98.50</td><td colspan="2">99.13</td><td colspan="2">93.30</td><td colspan="2">95.00</td></tr>
<tr><td>RVG</td><td colspan="2">-</td><td colspan="2">-</td><td colspan="2">57.77</td><td colspan="2">40.57</td></tr>
<tr><td rowspan="2">5</td><td rowspan="2">NSP1*</td><td>RVA</td><td colspan="2">98.57</td><td colspan="2">99.30</td><td colspan="2">94.22</td><td colspan="2">97.81</td></tr>
<tr><td>RVG</td><td colspan="2">-</td><td colspan="2">-</td><td>88.37</td><td>87.80</td><td>90.63</td><td>93.90</td></tr>
<tr><td rowspan="2">6</td><td rowspan="2">VP6</td><td>RVA</td><td colspan="2">99.37</td><td colspan="2">99.80</td><td colspan="2">93.97</td><td colspan="2">99.30</td></tr>
<tr><td>RVG</td><td colspan="2">-</td><td colspan="2">-</td><td colspan="2">90.10</td><td colspan="2">98.67</td></tr>
<tr><td rowspan="2">7</td><td rowspan="2">NSP3</td><td>RVA</td><td colspan="2">99.67</td><td colspan="2">99.53</td><td colspan="2">93.42</td><td colspan="2">96.42</td></tr>
<tr><td>RVG</td><td colspan="2">-</td><td colspan="2">-</td><td colspan="2">89.13</td><td colspan="2">92.13</td></tr>
<tr><td rowspan="2">8</td><td rowspan="2">NSP2</td><td>RVA</td><td colspan="2">99.60</td><td colspan="2">99.40</td><td colspan="2">95.76</td><td colspan="2">97.81</td></tr>
<tr><td>RVG</td><td colspan="2">-</td><td colspan="2">-</td><td colspan="2">90.07</td><td colspan="2">95.13</td></tr>
<tr><td rowspan="2">9</td><td rowspan="2">VP7</td><td>RVA</td><td colspan="2">99.33</td><td colspan="2">99.40</td><td colspan="2">93.01</td><td colspan="2">94.49</td></tr>
<tr><td>RVG</td><td colspan="2">-</td><td colspan="2">-</td><td colspan="2">67.70</td><td colspan="2">67.63</td></tr>
<tr><td rowspan="2">10</td><td rowspan="2">NSP4</td><td>RVA</td><td colspan="2">99.73</td><td colspan="2">100.00</td><td colspan="2">92.92</td><td colspan="2">94.75</td></tr>
<tr><td>RVG</td><td colspan="2">-</td><td colspan="2">-</td><td colspan="2">79.85</td><td colspan="2">74.90</td></tr>
<tr><td rowspan="2">11</td><td rowspan="2">NSP5</td><td>RVA</td><td colspan="2">99.40</td><td colspan="2">98.47</td><td colspan="2">93.54</td><td colspan="2">91.07</td></tr>
<tr><td>RVG</td><td colspan="2">-</td><td colspan="2">-</td><td colspan="2">83.27</td><td colspan="2">85.90</td></tr>
</table>
