# Supplementary material for: Rotaviruses in Pigeons With Diarrhea: Recovery of Three Complete Pigeon Rotavirus A Genomes and the First Case of Pigeon Rotavirus G in Europe
Source: Transbound Emerg Dis. 2024 Nov 25;2024:4684235. doi: 10.1155/tbed/4684235 (PMC12019971; doi:10.1155/tbed/4684235)
Supplement: Supporting Information 2 — Phylogenetic trees of nucleotide sequences of Rotavirus A genome segments: VP1 (a), VP2 (b), VP3 (c), VP4 (d), NSP1 (e), NSP3 (f), NSP2 (g), VP7 (h), NSP4 (i), and NSP5 (j). The trees consist of sequences obtained in this study and complete avian RVA sequences acquired from GenBank database. All sequences are labeled with the accession number and host name, country and year of collection as well as simplified genotype designation, while the sequences obtained in this study are labeled with the accession number and strain name and written in bold. Each tree is rooted with the sequence of the corresponding genome segment of pig rotavirus A isolate RVA/Pig/China/SC11/2017/G9P [23]. The trees were inferred in IQ-TREE 1.6.12 software [39, 40] and visualized with iTOL v6 software [41]. The distances were calculated with the maximum likelihood method with 1,000 bootstrap replicates. The substitution models most appropriate for each alignment were calculated with Find DNA/protein models tool in MEGA 11 software [38] and are as follows: GTR+G+I for VP1, VP2, VP3, and VP4 sequences, GTR+G for NSP1, NSP3, NSP2, and VP7 sequences and HKY+G for NSP4 and NSP5 sequences. [file 4684235.f2.pdf]

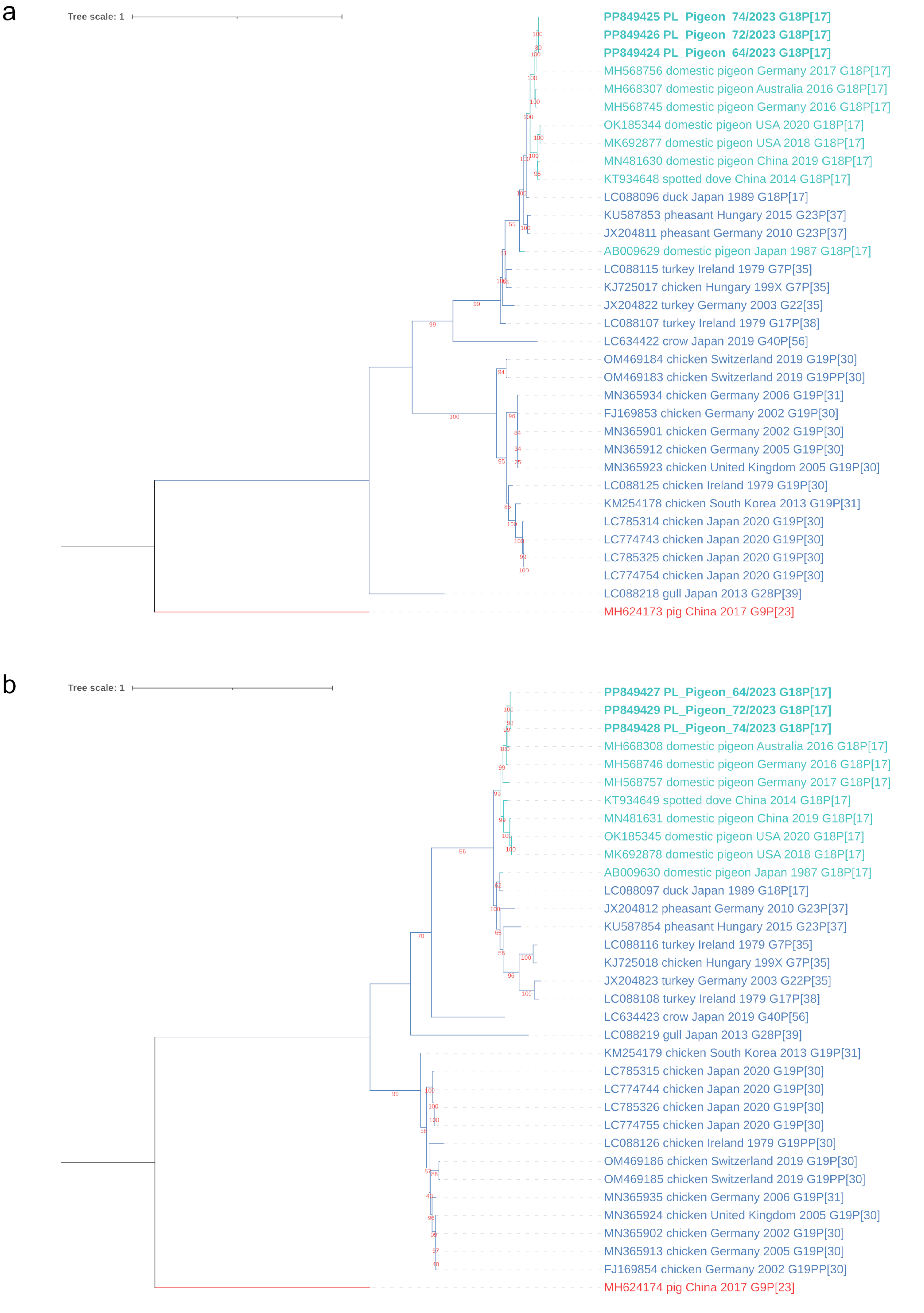
a
Tree scale: 1
PP849425 PL_Pigeon_74/2023 G18P[17]
PP849426 PL_Pigeon_72/2023 G18P[17]
PP849424 PL_Pigeon_64/2023 G18P[17]
MH568756 domestic pigeon Germany 2017 G18P[17]
MH668307 domestic pigeon Australia 2016 G18P[17]
MH568745 domestic pigeon Germany 2016 G18P[17]
OK185344 domestic pigeon USA 2020 G18P[17]
MK692877 domestic pigeon USA 2018 G18P[17]
MN481630 domestic pigeon China 2019 G18P[17]
KT934648 spotted dove China 2014 G18P[17]
LC088096 duck Japan 1989 G18P[17]
KU587853 pheasant Hungary 2015 G23P[37]
JX204811 pheasant Germany 2010 G23P[37]
AB009629 domestic pigeon Japan 1987 G18P[17]
LC088115 turkey Ireland 1979 G7P[35]
KJ725017 chicken Hungary 199X G7P[35]
JX204822 turkey Germany 2003 G22[35]
LC088107 turkey Ireland 1979 G17P[38]
LC634422 crow Japan 2019 G40P[56]
OM469184 chicken Switzerland 2019 G19P[30]
OM469183 chicken Switzerland 2019 G19PP[30]
MN365934 chicken Germany 2006 G19P[31]
FJ169853 chicken Germany 2002 G19P[30]
MN365901 chicken Germany 2002 G19P[30]
MN365912 chicken Germany 2005 G19P[30]
MN365923 chicken United Kingdom 2005 G19P[30]
LC088125 chicken Ireland 1979 G19P[30]
KM254178 chicken South Korea 2013 G19P[31]
LC785314 chicken Japan 2020 G19P[30]
LC774743 chicken Japan 2020 G19P[30]
LC785325 chicken Japan 2020 G19P[30]
LC774754 chicken Japan 2020 G19P[30]
LC088218 gull Japan 2013 G28P[39]
MH624173 pig China 2017 G9P[23]
b
Tree scale: 1
PP849427 PL_Pigeon_64/2023 G18P[17]
PP849429 PL_Pigeon_72/2023 G18P[17]
PP849428 PL_Pigeon_74/2023 G18P[17]
MH668308 domestic pigeon Australia 2016 G18P[17]
MH568746 domestic pigeon Germany 2016 G18P[17]
MH568757 domestic pigeon Germany 2017 G18P[17]
KT934649 spotted dove China 2014 G18P[17]
MN481631 domestic pigeon China 2019 G18P[17]
OK185345 domestic pigeon USA 2020 G18P[17]
MK692878 domestic pigeon USA 2018 G18P[17]
AB009630 domestic pigeon Japan 1987 G18P[17]
LC088097 duck Japan 1989 G18P[17]
JX204812 pheasant Germany 2010 G23P[37]
KU587854 pheasant Hungary 2015 G23P[37]
LC088116 turkey Ireland 1979 G7P[35]
KJ725018 chicken Hungary 199X G7P[35]
JX204823 turkey Germany 2003 G22P[35]
LC088108 turkey Ireland 1979 G17P[38]
LC634423 crow Japan 2019 G40P[56]
LC088219 gull Japan 2013 G28P[39]
KM254179 chicken South Korea 2013 G19P[31]
LC785315 chicken Japan 2020 G19P[30]
LC774744 chicken Japan 2020 G19P[30]
LC785326 chicken Japan 2020 G19P[30]
LC774755 chicken Japan 2020 G19P[30]
LC088126 chicken Ireland 1979 G19PP[30]
OM469186 chicken Switzerland 2019 G19P[30]
OM469185 chicken Switzerland 2019 G19PP[30]
MN365935 chicken Germany 2006 G19P[31]
MN365924 chicken United Kingdom 2005 G19P[30]
MN365902 chicken Germany 2002 G19P[30]
MN365913 chicken Germany 2005 G19P[30]
FJ169854 chicken Germany 2002 G19PP[30]
MH624174 pig China 2017 G9P[23]

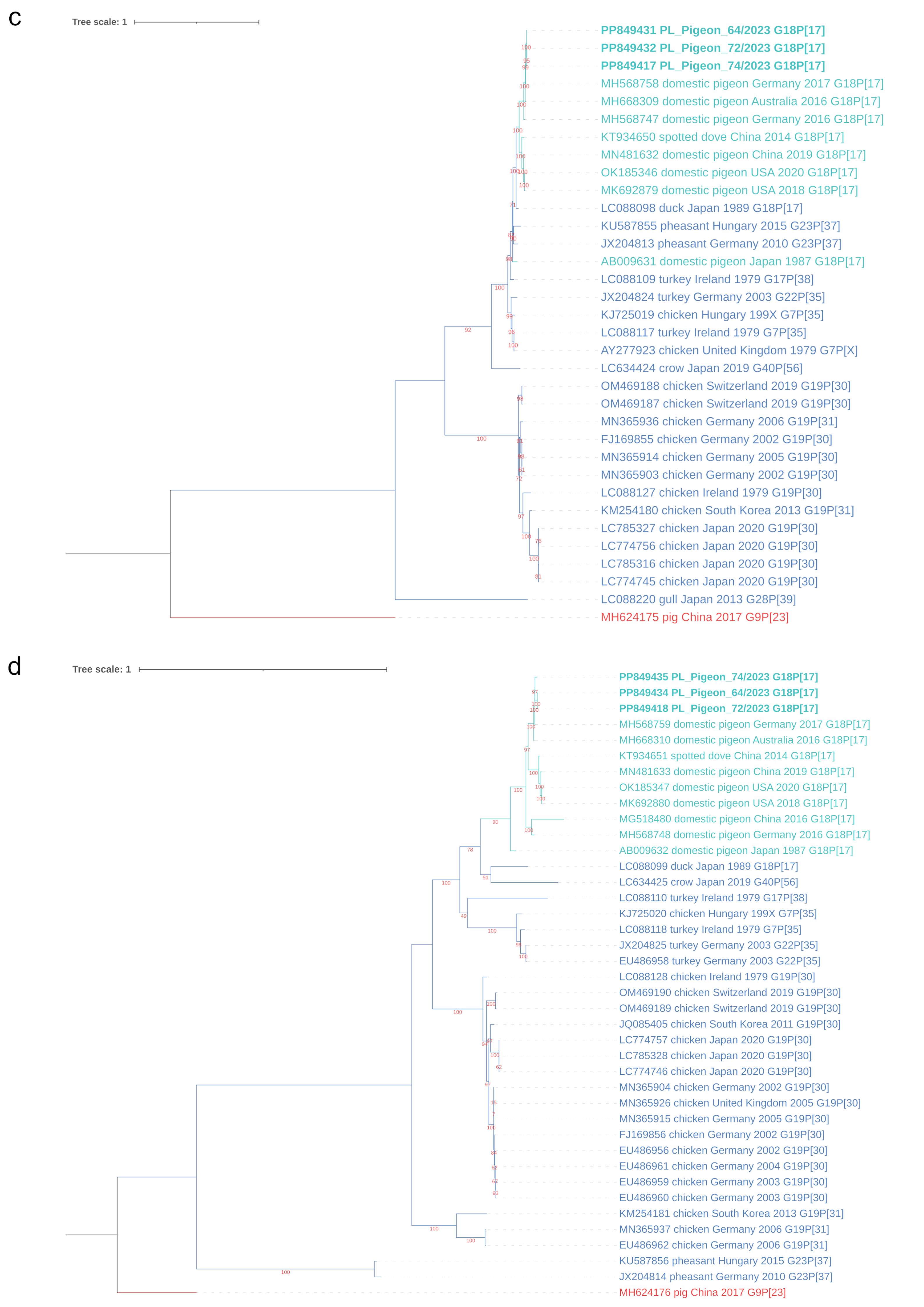
c
Tree scale: 1
PP849431 PL_Pigeon_64/2023 G18P[17]
PP849432 PL_Pigeon_72/2023 G18P[17]
PP849417 PL_Pigeon_74/2023 G18P[17]
MH568758 domestic pigeon Germany 2017 G18P[17]
MH668309 domestic pigeon Australia 2016 G18P[17]
MH568747 domestic pigeon Germany 2016 G18P[17]
KT934650 spotted dove China 2014 G18P[17]
MN481632 domestic pigeon China 2019 G18P[17]
OK185346 domestic pigeon USA 2020 G18P[17]
MK692879 domestic pigeon USA 2018 G18P[17]
LC088098 duck Japan 1989 G18P[17]
KU587855 pheasant Hungary 2015 G23P[37]
JX204813 pheasant Germany 2010 G23P[37]
AB009631 domestic pigeon Japan 1987 G18P[17]
LC088109 turkey Ireland 1979 G17P[38]
JX204824 turkey Germany 2003 G22P[35]
KJ725019 chicken Hungary 199X G7P[35]
LC088117 turkey Ireland 1979 G7P[35]
AY277923 chicken United Kingdom 1979 G7P[X]
LC634424 crow Japan 2019 G40P[56]
OM469188 chicken Switzerland 2019 G19P[30]
OM469187 chicken Switzerland 2019 G19P[30]
MN365936 chicken Germany 2006 G19P[31]
FJ169855 chicken Germany 2002 G19P[30]
MN365914 chicken Germany 2005 G19P[30]
MN365903 chicken Germany 2002 G19P[30]
LC088127 chicken Ireland 1979 G19P[30]
KM254180 chicken South Korea 2013 G19P[31]
LC785327 chicken Japan 2020 G19P[30]
LC774756 chicken Japan 2020 G19P[30]
LC785316 chicken Japan 2020 G19P[30]
LC774745 chicken Japan 2020 G19P[30]
LC088220 gull Japan 2013 G28P[39]
MH624175 pig China 2017 G9P[23]
d
Tree scale: 1
PP849435 PL_Pigeon_74/2023 G18P[17]
PP849434 PL_Pigeon_64/2023 G18P[17]
PP849418 PL_Pigeon_72/2023 G18P[17]
MH568759 domestic pigeon Germany 2017 G18P[17]
MH668310 domestic pigeon Australia 2016 G18P[17]
KT934651 spotted dove China 2014 G18P[17]
MN481633 domestic pigeon China 2019 G18P[17]
OK185347 domestic pigeon USA 2020 G18P[17]
MK692880 domestic pigeon USA 2018 G18P[17]
MG518480 domestic pigeon China 2016 G18P[17]
MH568748 domestic pigeon Germany 2016 G18P[17]
AB009632 domestic pigeon Japan 1987 G18P[17]
LC088099 duck Japan 1989 G18P[17]
LC634425 crow Japan 2019 G40P[56]
LC088110 turkey Ireland 1979 G17P[38]
KJ725020 chicken Hungary 199X G7P[35]
LC088118 turkey Ireland 1979 G7P[35]
JX204825 turkey Germany 2003 G22P[35]
EU486958 turkey Germany 2003 G22P[35]
LC088128 chicken Ireland 1979 G19P[30]
OM469190 chicken Switzerland 2019 G19P[30]
OM469189 chicken Switzerland 2019 G19P[30]
JQ085405 chicken South Korea 2011 G19P[30]
LC774757 chicken Japan 2020 G19P[30]
LC785328 chicken Japan 2020 G19P[30]
LC774746 chicken Japan 2020 G19P[30]
MN365904 chicken Germany 2002 G19P[30]
MN365926 chicken United Kingdom 2005 G19P[30]
MN365915 chicken Germany 2005 G19P[30]
FJ169856 chicken Germany 2002 G19P[30]
EU486956 chicken Germany 2002 G19P[30]
EU486961 chicken Germany 2004 G19P[30]
EU486959 chicken Germany 2003 G19P[30]
EU486960 chicken Germany 2003 G19P[30]
KM254181 chicken South Korea 2013 G19P[31]
MN365937 chicken Germany 2006 G19P[31]
EU486962 chicken Germany 2006 G19P[31]
KU587856 pheasant Hungary 2015 G23P[37]
JX204814 pheasant Germany 2010 G23P[37]
MH624176 pig China 2017 G9P[23]

e

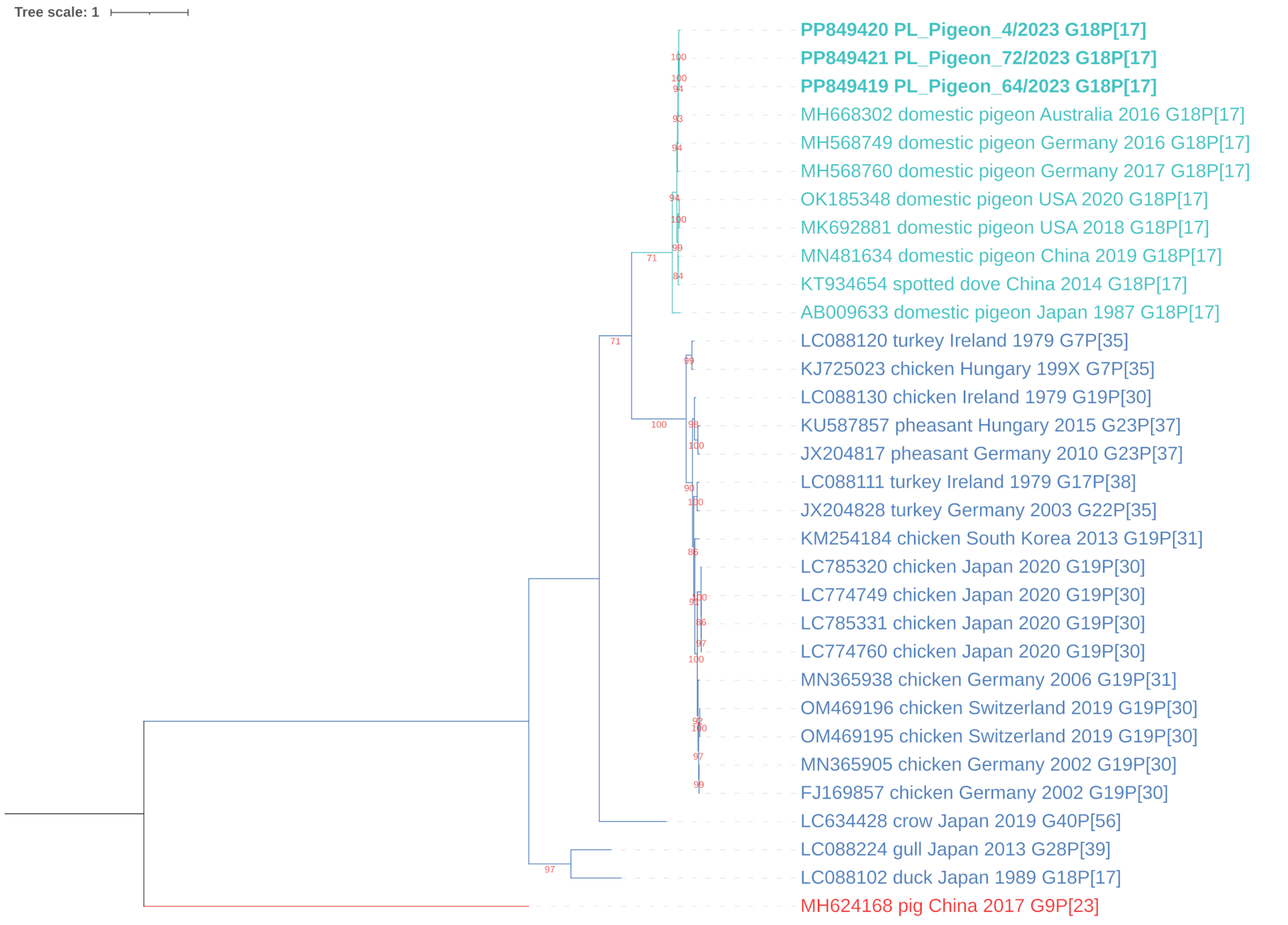
Tree scale: 1
PP849420 PL_Pigeon_4/2023 G18P[17]
PP849421 PL_Pigeon_72/2023 G18P[17]
PP849419 PL_Pigeon_64/2023 G18P[17]
MH668302 domestic pigeon Australia 2016 G18P[17]
MH568749 domestic pigeon Germany 2016 G18P[17]
MH568760 domestic pigeon Germany 2017 G18P[17]
OK185348 domestic pigeon USA 2020 G18P[17]
MK692881 domestic pigeon USA 2018 G18P[17]
MN481634 domestic pigeon China 2019 G18P[17]
KT934654 spotted dove China 2014 G18P[17]
AB009633 domestic pigeon Japan 1987 G18P[17]
LC088120 turkey Ireland 1979 G7P[35]
KJ725023 chicken Hungary 199X G7P[35]
LC088130 chicken Ireland 1979 G19P[30]
KU587857 pheasant Hungary 2015 G23P[37]
JX204817 pheasant Germany 2010 G23P[37]
LC088111 turkey Ireland 1979 G17P[38]
JX204828 turkey Germany 2003 G22P[35]
KM254184 chicken South Korea 2013 G19P[31]
LC785320 chicken Japan 2020 G19P[30]
LC774749 chicken Japan 2020 G19P[30]
LC785331 chicken Japan 2020 G19P[30]
LC774760 chicken Japan 2020 G19P[30]
MN365938 chicken Germany 2006 G19P[31]
OM469196 chicken Switzerland 2019 G19P[30]
OM469195 chicken Switzerland 2019 G19P[30]
MN365905 chicken Germany 2002 G19P[30]
FJ169857 chicken Germany 2002 G19P[30]
LC634428 crow Japan 2019 G40P[56]
LC088224 gull Japan 2013 G28P[39]
LC088102 duck Japan 1989 G18P[17]
MH624168 pig China 2017 G9P[23]

f

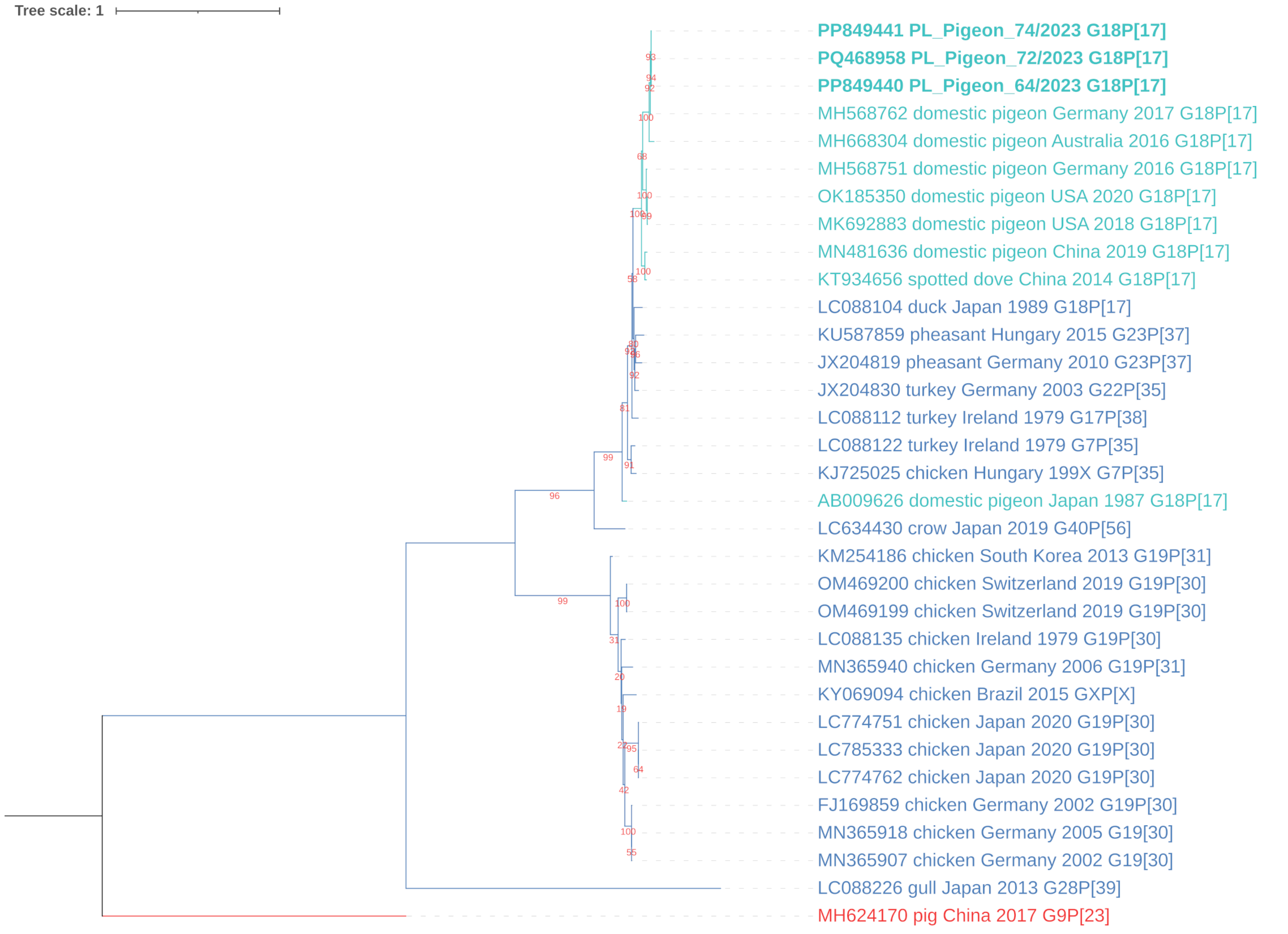
Tree scale: 1
PP849441 PL_Pigeon_74/2023 G18P[17]
PQ468958 PL_Pigeon_72/2023 G18P[17]
PP849440 PL_Pigeon_64/2023 G18P[17]
MH568762 domestic pigeon Germany 2017 G18P[17]
MH668304 domestic pigeon Australia 2016 G18P[17]
MH568751 domestic pigeon Germany 2016 G18P[17]
OK185350 domestic pigeon USA 2020 G18P[17]
MK692883 domestic pigeon USA 2018 G18P[17]
MN481636 domestic pigeon China 2019 G18P[17]
KT934656 spotted dove China 2014 G18P[17]
LC088104 duck Japan 1989 G18P[17]
KU587859 pheasant Hungary 2015 G23P[37]
JX204819 pheasant Germany 2010 G23P[37]
JX204830 turkey Germany 2003 G22P[35]
LC088112 turkey Ireland 1979 G17P[38]
LC088122 turkey Ireland 1979 G7P[35]
KJ725025 chicken Hungary 199X G7P[35]
AB009626 domestic pigeon Japan 1987 G18P[17]
LC634430 crow Japan 2019 G40P[56]
KM254186 chicken South Korea 2013 G19P[31]
OM469200 chicken Switzerland 2019 G19P[30]
OM469199 chicken Switzerland 2019 G19P[30]
LC088135 chicken Ireland 1979 G19P[30]
MN365940 chicken Germany 2006 G19P[31]
KY069094 chicken Brazil 2015 GXP[X]
LC774751 chicken Japan 2020 G19P[30]
LC785333 chicken Japan 2020 G19P[30]
LC774762 chicken Japan 2020 G19P[30]
FJ169859 chicken Germany 2002 G19P[30]
MN365918 chicken Germany 2005 G19[30]
MN365907 chicken Germany 2002 G19[30]
LC088226 gull Japan 2013 G28P[39]
MH624170 pig China 2017 G9P[23]

g

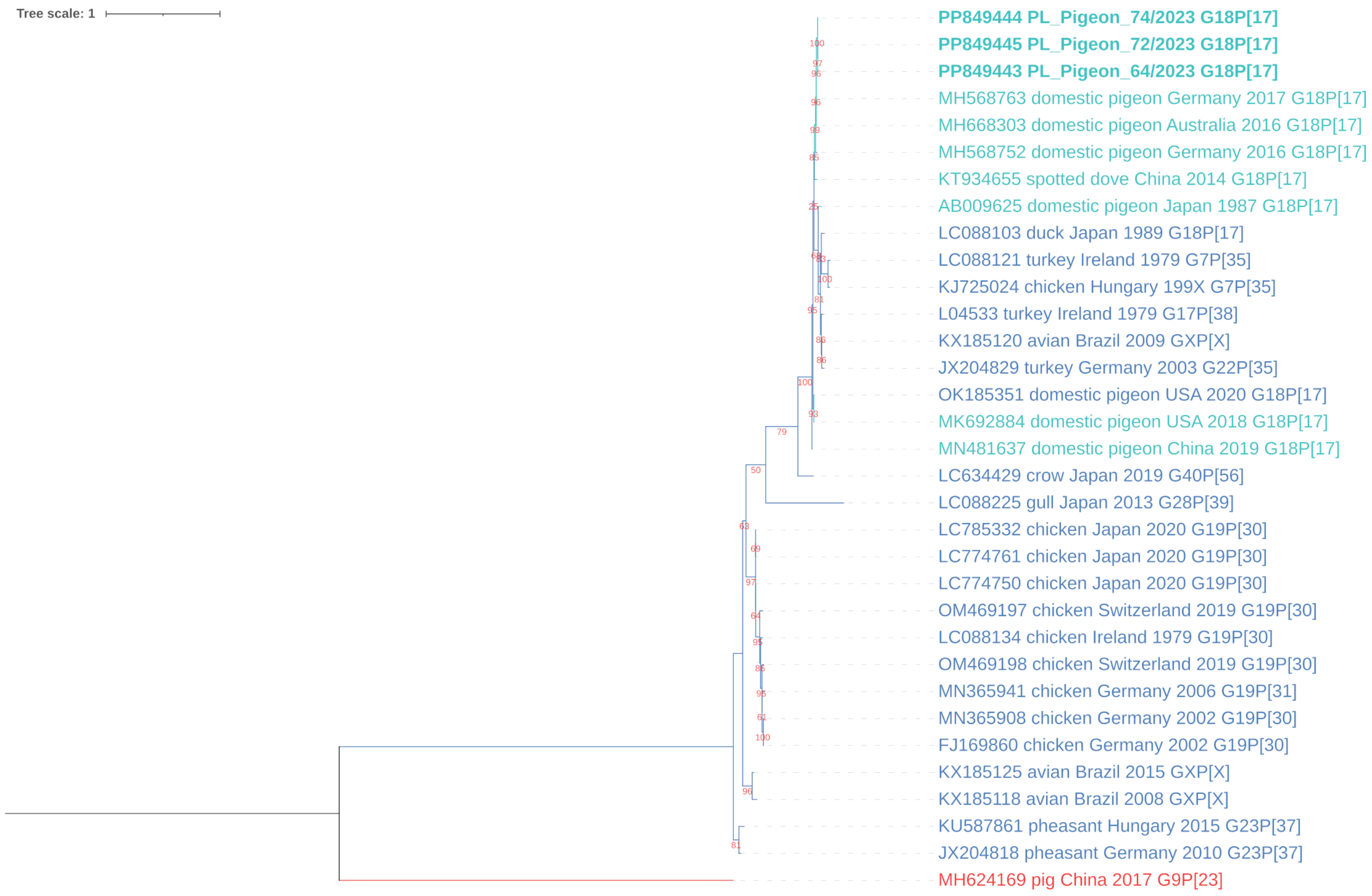
Tree scale: 1
PP849444 PL_Pigeon_74/2023 G18P[17]
PP849445 PL_Pigeon_72/2023 G18P[17]
PP849443 PL_Pigeon_64/2023 G18P[17]
MH568763 domestic pigeon Germany 2017 G18P[17]
MH668303 domestic pigeon Australia 2016 G18P[17]
MH568752 domestic pigeon Germany 2016 G18P[17]
KT934655 spotted dove China 2014 G18P[17]
AB009625 domestic pigeon Japan 1987 G18P[17]
LC088103 duck Japan 1989 G18P[17]
LC088121 turkey Ireland 1979 G7P[35]
KJ725024 chicken Hungary 199X G7P[35]
L04533 turkey Ireland 1979 G17P[38]
KX185120 avian Brazil 2009 GXP[X]
JX204829 turkey Germany 2003 G22P[35]
OK185351 domestic pigeon USA 2020 G18P[17]
MK692884 domestic pigeon USA 2018 G18P[17]
MN481637 domestic pigeon China 2019 G18P[17]
LC634429 crow Japan 2019 G40P[56]
LC088225 gull Japan 2013 G28P[39]
LC785332 chicken Japan 2020 G19P[30]
LC774761 chicken Japan 2020 G19P[30]
LC774750 chicken Japan 2020 G19P[30]
OM469197 chicken Switzerland 2019 G19P[30]
LC088134 chicken Ireland 1979 G19P[30]
OM469198 chicken Switzerland 2019 G19P[30]
MN365941 chicken Germany 2006 G19P[31]
MN365908 chicken Germany 2002 G19P[30]
FJ169860 chicken Germany 2002 G19P[30]
KX185125 avian Brazil 2015 GXP[X]
KX185118 avian Brazil 2008 GXP[X]
KU587861 pheasant Hungary 2015 G23P[37]
JX204818 pheasant Germany 2010 G23P[37]
MH624169 pig China 2017 G9P[23]

h

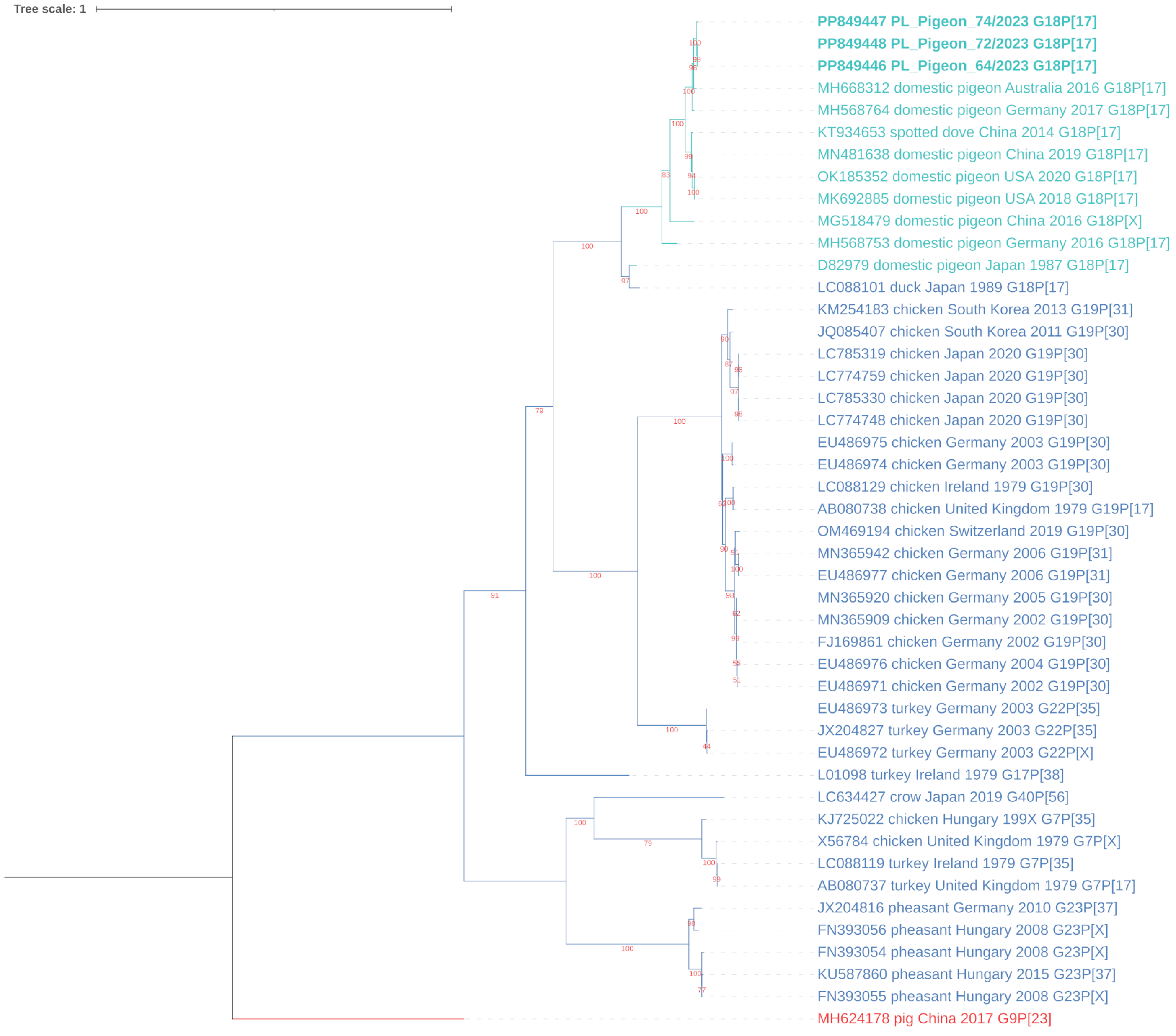
Tree scale: 1
PP849447 PL_Pigeon_74/2023 G18P[17]
PP849448 PL_Pigeon_72/2023 G18P[17]
PP849446 PL_Pigeon_64/2023 G18P[17]
MH668312 domestic pigeon Australia 2016 G18P[17]
MH568764 domestic pigeon Germany 2017 G18P[17]
KT934653 spotted dove China 2014 G18P[17]
MN481638 domestic pigeon China 2019 G18P[17]
OK185352 domestic pigeon USA 2020 G18P[17]
MK692885 domestic pigeon USA 2018 G18P[17]
MG518479 domestic pigeon China 2016 G18P[X]
MH568753 domestic pigeon Germany 2016 G18P[17]
D82979 domestic pigeon Japan 1987 G18P[17]
LC088101 duck Japan 1989 G18P[17]
KM254183 chicken South Korea 2013 G19P[31]
JQ085407 chicken South Korea 2011 G19P[30]
LC785319 chicken Japan 2020 G19P[30]
LC774759 chicken Japan 2020 G19P[30]
LC785330 chicken Japan 2020 G19P[30]
LC774748 chicken Japan 2020 G19P[30]
EU486975 chicken Germany 2003 G19P[30]
EU486974 chicken Germany 2003 G19P[30]
LC088129 chicken Ireland 1979 G19P[30]
AB080738 chicken United Kingdom 1979 G19P[17]
OM469194 chicken Switzerland 2019 G19P[30]
MN365942 chicken Germany 2006 G19P[31]
EU486977 chicken Germany 2006 G19P[31]
MN365920 chicken Germany 2005 G19P[30]
MN365909 chicken Germany 2002 G19P[30]
FJ169861 chicken Germany 2002 G19P[30]
EU486976 chicken Germany 2004 G19P[30]
EU486971 chicken Germany 2002 G19P[30]
EU486973 turkey Germany 2003 G22P[35]
JX204827 turkey Germany 2003 G22P[35]
EU486972 turkey Germany 2003 G22P[X]
L01098 turkey Ireland 1979 G17P[38]
LC634427 crow Japan 2019 G40P[56]
KJ725022 chicken Hungary 199X G7P[35]
X56784 chicken United Kingdom 1979 G7P[X]
LC088119 turkey Ireland 1979 G7P[35]
AB080737 turkey United Kingdom 1979 G7P[17]
JX204816 pheasant Germany 2010 G23P[37]
FN393056 pheasant Hungary 2008 G23P[X]
FN393054 pheasant Hungary 2008 G23P[X]
KU587860 pheasant Hungary 2015 G23P[37]
FN393055 pheasant Hungary 2008 G23P[X]
MH624178 pig China 2017 G9P[23]

i

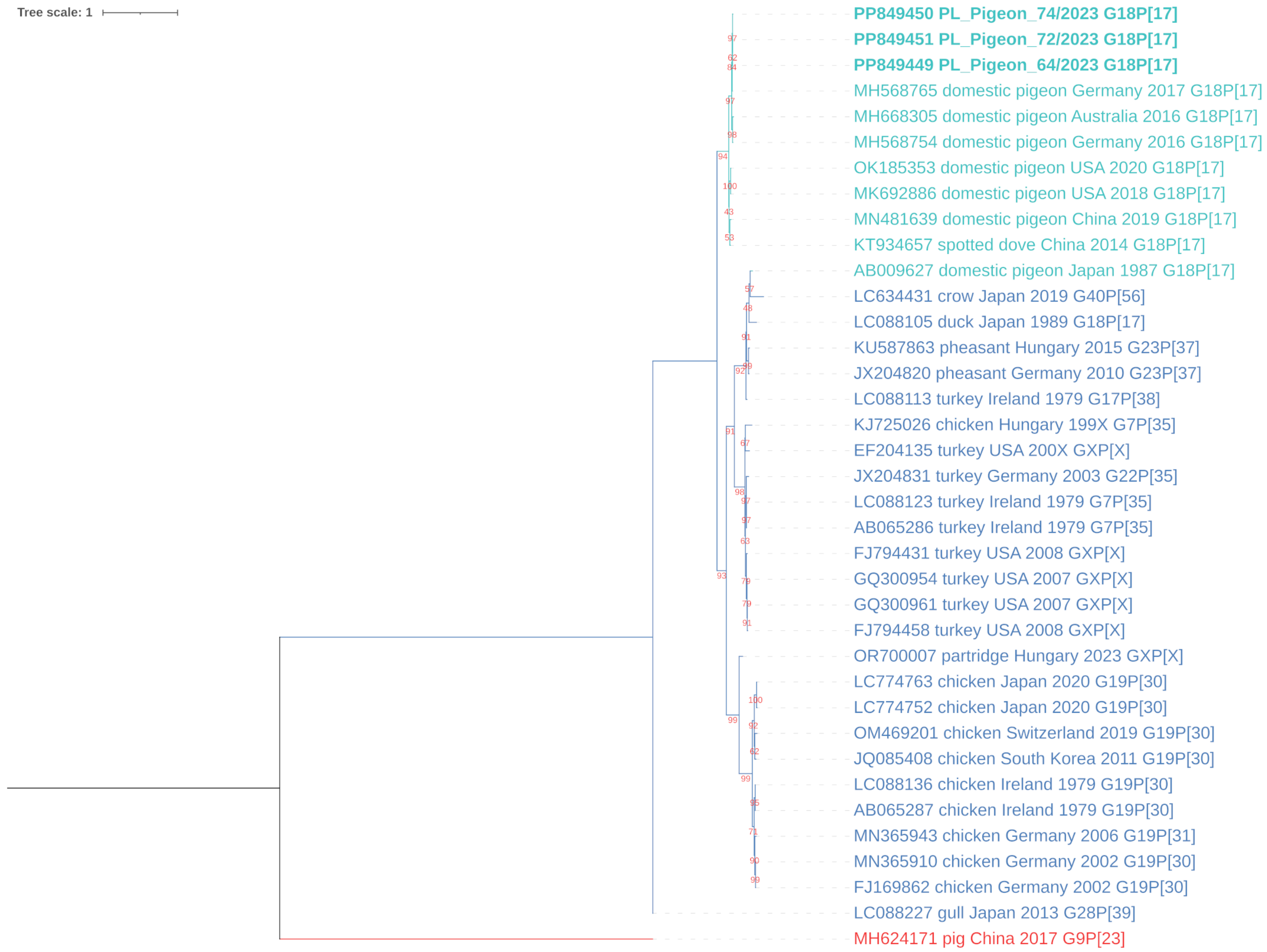
Tree scale: 1
PP849450 PL_Pigeon_74/2023 G18P[17]
PP849451 PL_Pigeon_72/2023 G18P[17]
PP849449 PL_Pigeon_64/2023 G18P[17]
MH568765 domestic pigeon Germany 2017 G18P[17]
MH668305 domestic pigeon Australia 2016 G18P[17]
MH568754 domestic pigeon Germany 2016 G18P[17]
OK185353 domestic pigeon USA 2020 G18P[17]
MK692886 domestic pigeon USA 2018 G18P[17]
MN481639 domestic pigeon China 2019 G18P[17]
KT934657 spotted dove China 2014 G18P[17]
AB009627 domestic pigeon Japan 1987 G18P[17]
LC634431 crow Japan 2019 G40P[56]
LC088105 duck Japan 1989 G18P[17]
KU587863 pheasant Hungary 2015 G23P[37]
JX204820 pheasant Germany 2010 G23P[37]
LC088113 turkey Ireland 1979 G17P[38]
KJ725026 chicken Hungary 199X G7P[35]
EF204135 turkey USA 200X GXP[X]
JX204831 turkey Germany 2003 G22P[35]
LC088123 turkey Ireland 1979 G7P[35]
AB065286 turkey Ireland 1979 G7P[35]
FJ794431 turkey USA 2008 GXP[X]
GQ300954 turkey USA 2007 GXP[X]
GQ300961 turkey USA 2007 GXP[X]
FJ794458 turkey USA 2008 GXP[X]
OR700007 partridge Hungary 2023 GXP[X]
LC774763 chicken Japan 2020 G19P[30]
LC774752 chicken Japan 2020 G19P[30]
OM469201 chicken Switzerland 2019 G19P[30]
JQ085408 chicken South Korea 2011 G19P[30]
LC088136 chicken Ireland 1979 G19P[30]
AB065287 chicken Ireland 1979 G19P[30]
MN365943 chicken Germany 2006 G19P[31]
MN365910 chicken Germany 2002 G19P[30]
FJ169862 chicken Germany 2002 G19P[30]
LC088227 gull Japan 2013 G28P[39]
MH624171 pig China 2017 G9P[23]

j

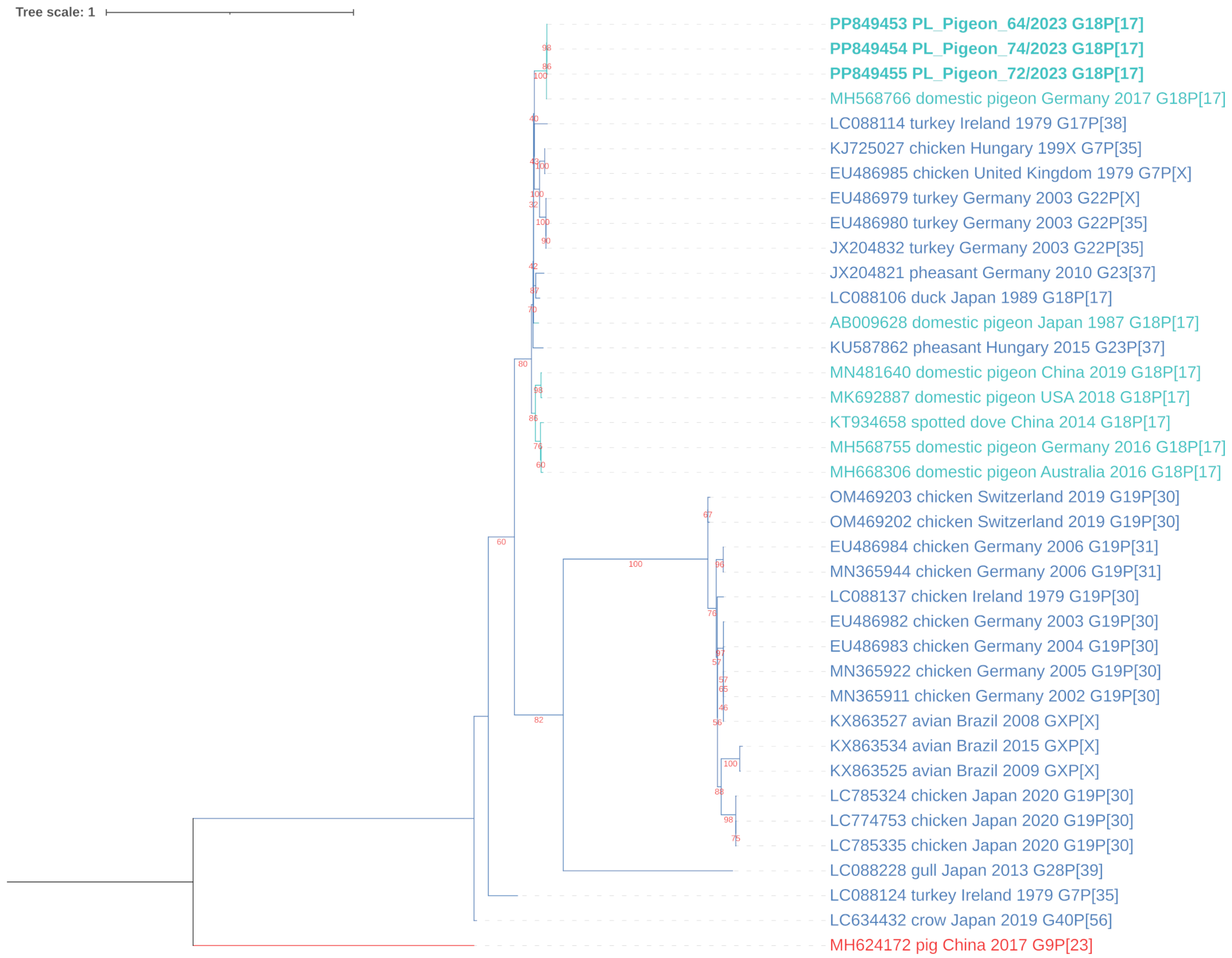
Tree scale: 1
PP849453 PL_Pigeon_64/2023 G18P[17]
PP849454 PL_Pigeon_74/2023 G18P[17]
PP849455 PL_Pigeon_72/2023 G18P[17]
MH568766 domestic pigeon Germany 2017 G18P[17]
LC088114 turkey Ireland 1979 G17P[38]
KJ725027 chicken Hungary 199X G7P[35]
EU486985 chicken United Kingdom 1979 G7P[X]
EU486979 turkey Germany 2003 G22P[X]
EU486980 turkey Germany 2003 G22P[35]
JX204832 turkey Germany 2003 G22P[35]
JX204821 pheasant Germany 2010 G23[37]
LC088106 duck Japan 1989 G18P[17]
AB009628 domestic pigeon Japan 1987 G18P[17]
KU587862 pheasant Hungary 2015 G23P[37]
MN481640 domestic pigeon China 2019 G18P[17]
MK692887 domestic pigeon USA 2018 G18P[17]
KT934658 spotted dove China 2014 G18P[17]
MH568755 domestic pigeon Germany 2016 G18P[17]
MH668306 domestic pigeon Australia 2016 G18P[17]
OM469203 chicken Switzerland 2019 G19P[30]
OM469202 chicken Switzerland 2019 G19P[30]
EU486984 chicken Germany 2006 G19P[31]
MN365944 chicken Germany 2006 G19P[31]
LC088137 chicken Ireland 1979 G19P[30]
EU486982 chicken Germany 2003 G19P[30]
EU486983 chicken Germany 2004 G19P[30]
MN365922 chicken Germany 2005 G19P[30]
MN365911 chicken Germany 2002 G19P[30]
KX863527 avian Brazil 2008 GXP[X]
KX863534 avian Brazil 2015 GXP[X]
KX863525 avian Brazil 2009 GXP[X]
LC785324 chicken Japan 2020 G19P[30]
LC774753 chicken Japan 2020 G19P[30]
LC785335 chicken Japan 2020 G19P[30]
LC088228 gull Japan 2013 G28P[39]
LC088124 turkey Ireland 1979 G7P[35]
LC634432 crow Japan 2019 G40P[56]
MH624172 pig China 2017 G9P[23]
