# Supplementary material for: Rotaviruses in Pigeons With Diarrhea: Recovery of Three Complete Pigeon Rotavirus A Genomes and the First Case of Pigeon Rotavirus G in Europe
Source: Transbound Emerg Dis. 2024 Nov 25;2024:4684235. doi: 10.1155/tbed/4684235 (PMC12019971; doi:10.1155/tbed/4684235)
Supplement: Supporting Information 3 — Phylogenetic trees of nucleotide sequences of Rotavirus G genome segments: VP1 (a), VP2 (b), VP3 (c), VP4 (d), NSP1-1 and NSP1-2 (e), NSP3 (f), NSP2 (g), VP7 (h), NSP4 (i), and NSP5 (j). The trees consist of sequences obtained in this study and complete RVG sequences acquired from GenBank database. All sequences are labeled with the accession number and host name as well as country and year of collection, while the sequences obtained in this study are labeled with the accession number and strain name and written in bold. Each tree is rooted with the sequence of the corresponding genome segment of avian rotavirus PO-13. The trees were inferred in IQ-TREE 1.6.12 software [39, 40] and visualized with iTOL v6 software [41]. The distances were calculated with the maximum likelihood method with 1000 bootstrap replicates. The substitution models most appropriate for each alignment were calculated with find DNA/protein models tool in MEGA 11 software [38] and are as follows: GTR+G for VP1, VP2, VP3, VP4, and VP7 sequences, TN93+G for NSP1-1 and NSP1-2, NSP3, NSP2, and NSP5 sequences and HKY+G for NSP4 sequences. [file 4684235.f3.pdf]

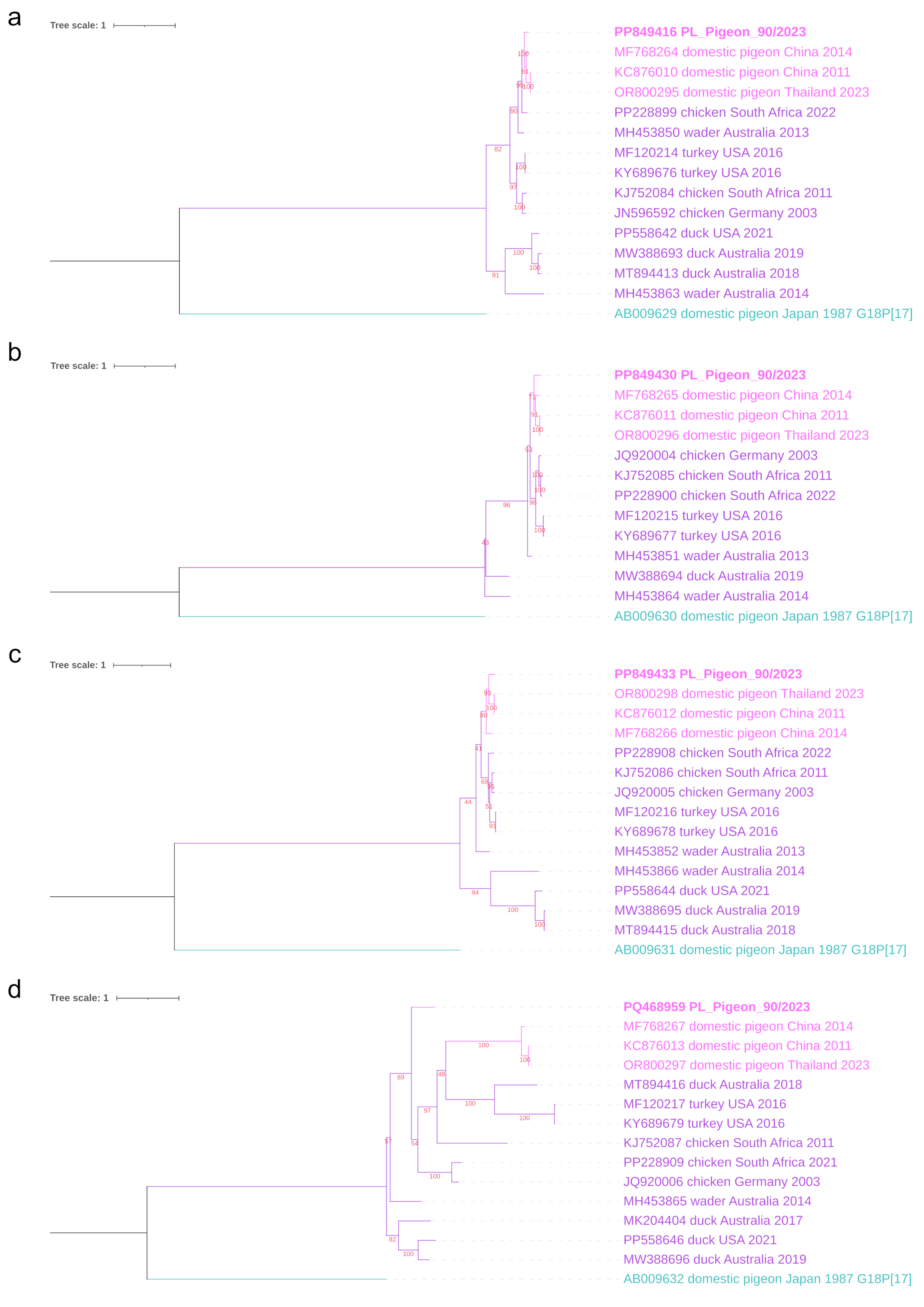
a
Tree scale: 1
PP849416 PL_Pigeon_90/2023
MF768264 domestic pigeon China 2014
KC876010 domestic pigeon China 2011
OR800295 domestic pigeon Thailand 2023
PP228899 chicken South Africa 2022
MH453850 wader Australia 2013
MF120214 turkey USA 2016
KY689676 turkey USA 2016
KJ752084 chicken South Africa 2011
JN596592 chicken Germany 2003
PP558642 duck USA 2021
MW388693 duck Australia 2019
MT894413 duck Australia 2018
MH453863 wader Australia 2014
AB009629 domestic pigeon Japan 1987 G18P[17]
b
Tree scale: 1
PP849430 PL_Pigeon_90/2023
MF768265 domestic pigeon China 2014
KC876011 domestic pigeon China 2011
OR800296 domestic pigeon Thailand 2023
JQ920004 chicken Germany 2003
KJ752085 chicken South Africa 2011
PP228900 chicken South Africa 2022
MF120215 turkey USA 2016
KY689677 turkey USA 2016
MH453851 wader Australia 2013
MW388694 duck Australia 2019
MH453864 wader Australia 2014
AB009630 domestic pigeon Japan 1987 G18P[17]
c
Tree scale: 1
PP849433 PL_Pigeon_90/2023
OR800298 domestic pigeon Thailand 2023
KC876012 domestic pigeon China 2011
MF768266 domestic pigeon China 2014
PP228908 chicken South Africa 2022
KJ752086 chicken South Africa 2011
JQ920005 chicken Germany 2003
MF120216 turkey USA 2016
KY689678 turkey USA 2016
MH453852 wader Australia 2013
MH453866 wader Australia 2014
PP558644 duck USA 2021
MW388695 duck Australia 2019
MT894415 duck Australia 2018
AB009631 domestic pigeon Japan 1987 G18P[17]
d
Tree scale: 1
PQ468959 PL_Pigeon_90/2023
MF768267 domestic pigeon China 2014
KC876013 domestic pigeon China 2011
OR800297 domestic pigeon Thailand 2023
MT894416 duck Australia 2018
MF120217 turkey USA 2016
KY689679 turkey USA 2016
KJ752087 chicken South Africa 2011
PP228909 chicken South Africa 2021
JQ920006 chicken Germany 2003
MH453865 wader Australia 2014
MK204404 duck Australia 2017
PP558646 duck USA 2021
MW388696 duck Australia 2019
AB009632 domestic pigeon Japan 1987 G18P[17]

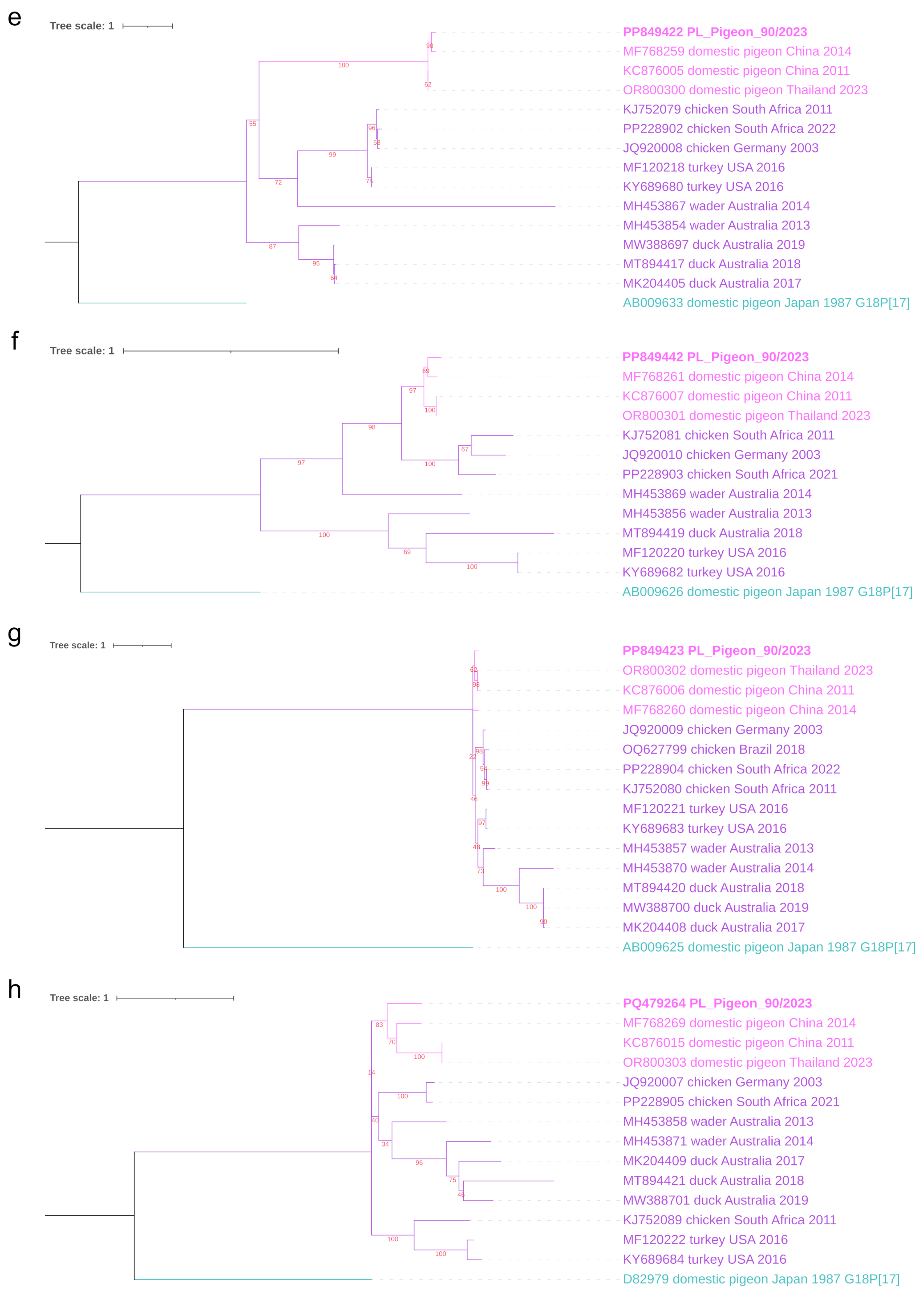

e
Tree scale: 1
PP849422 PL_Pigeon_90/2023
MF768259 domestic pigeon China 2014
KC876005 domestic pigeon China 2011
OR800300 domestic pigeon Thailand 2023
KJ752079 chicken South Africa 2011
PP228902 chicken South Africa 2022
JQ920008 chicken Germany 2003
MF120218 turkey USA 2016
KY689680 turkey USA 2016
MH453867 wader Australia 2014
MH453854 wader Australia 2013
MW388697 duck Australia 2019
MT894417 duck Australia 2018
MK204405 duck Australia 2017
AB009633 domestic pigeon Japan 1987 G18P[17]
f
Tree scale: 1
PP849442 PL_Pigeon_90/2023
MF768261 domestic pigeon China 2014
KC876007 domestic pigeon China 2011
OR800301 domestic pigeon Thailand 2023
KJ752081 chicken South Africa 2011
JQ920010 chicken Germany 2003
PP228903 chicken South Africa 2021
MH453869 wader Australia 2014
MH453856 wader Australia 2013
MT894419 duck Australia 2018
MF120220 turkey USA 2016
KY689682 turkey USA 2016
AB009626 domestic pigeon Japan 1987 G18P[17]
g
Tree scale: 1
PP849423 PL_Pigeon_90/2023
OR800302 domestic pigeon Thailand 2023
KC876006 domestic pigeon China 2011
MF768260 domestic pigeon China 2014
JQ920009 chicken Germany 2003
OQ627799 chicken Brazil 2018
PP228904 chicken South Africa 2022
KJ752080 chicken South Africa 2011
MF120221 turkey USA 2016
KY689683 turkey USA 2016
MH453857 wader Australia 2013
MH453870 wader Australia 2014
MT894420 duck Australia 2018
MW388700 duck Australia 2019
MK204408 duck Australia 2017
AB009625 domestic pigeon Japan 1987 G18P[17]
h
Tree scale: 1
PQ479264 PL_Pigeon_90/2023
MF768269 domestic pigeon China 2014
KC876015 domestic pigeon China 2011
OR800303 domestic pigeon Thailand 2023
JQ920007 chicken Germany 2003
PP228905 chicken South Africa 2021
MH453858 wader Australia 2013
MH453871 wader Australia 2014
MK204409 duck Australia 2017
MT894421 duck Australia 2018
MW388701 duck Australia 2019
KJ752089 chicken South Africa 2011
MF120222 turkey USA 2016
KY689684 turkey USA 2016
D82979 domestic pigeon Japan 1987 G18P[17]

i

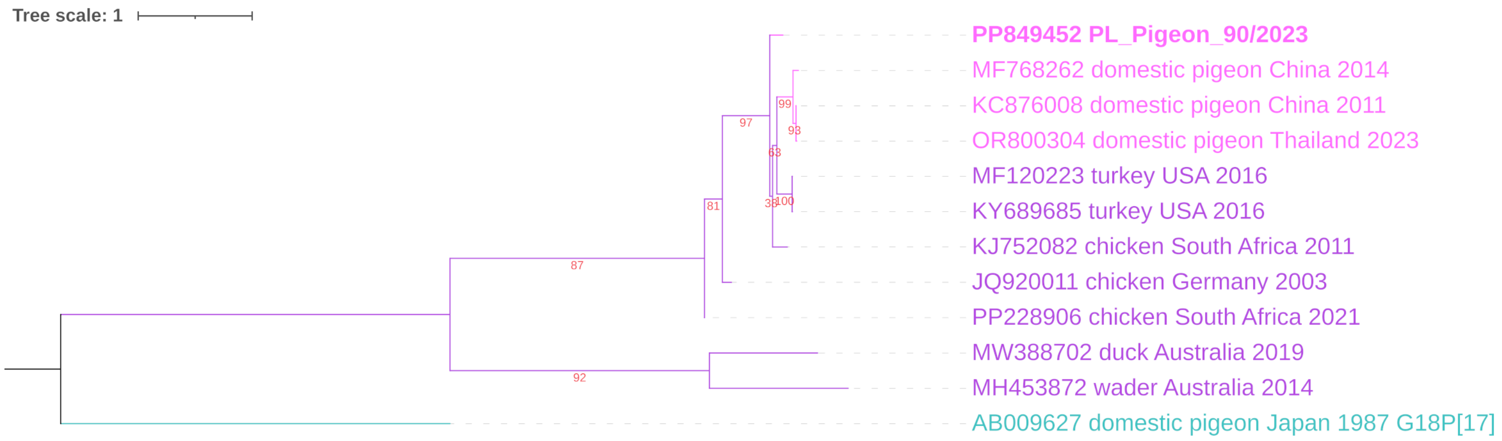
Tree scale: 1
PP849452 PL_Pigeon_90/2023
MF768262 domestic pigeon China 2014
KC876008 domestic pigeon China 2011
OR800304 domestic pigeon Thailand 2023
MF120223 turkey USA 2016
KY689685 turkey USA 2016
KJ752082 chicken South Africa 2011
JQ920011 chicken Germany 2003
PP228906 chicken South Africa 2021
MW388702 duck Australia 2019
MH453872 wader Australia 2014
AB009627 domestic pigeon Japan 1987 G18P[17]

j

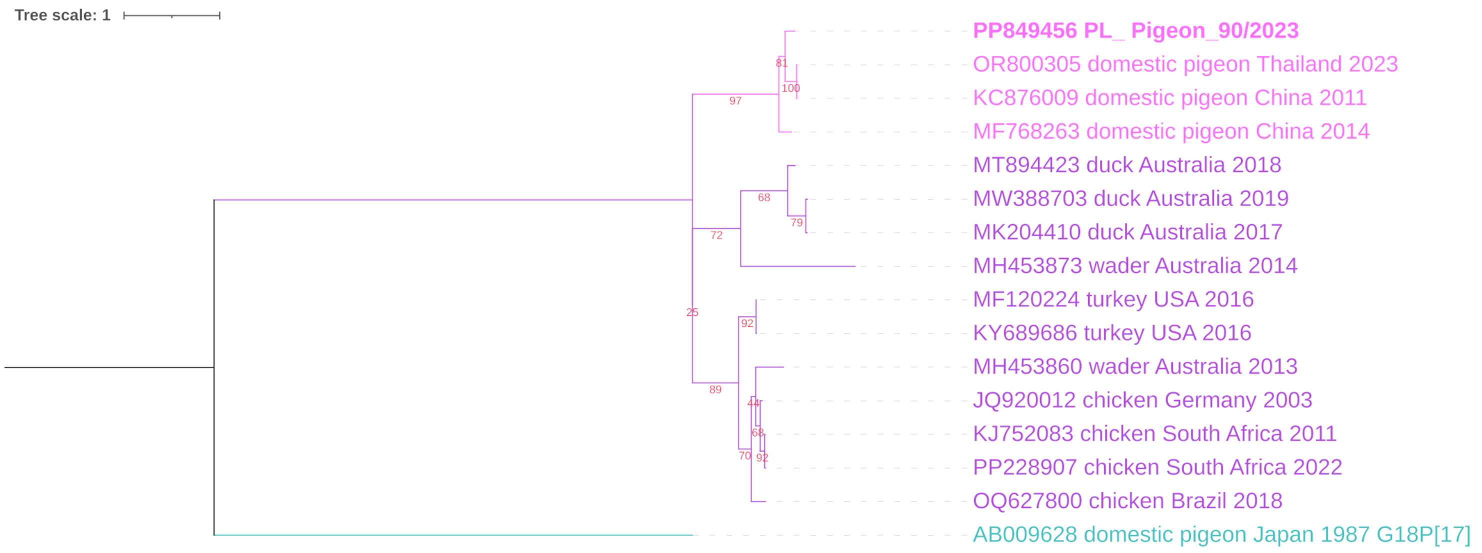
Tree scale: 1
PP849456 PL_ Pigeon_90/2023
OR800305 domestic pigeon Thailand 2023
KC876009 domestic pigeon China 2011
MF768263 domestic pigeon China 2014
MT894423 duck Australia 2018
MW388703 duck Australia 2019
MK204410 duck Australia 2017
MH453873 wader Australia 2014
MF120224 turkey USA 2016
KY689686 turkey USA 2016
MH453860 wader Australia 2013
JQ920012 chicken Germany 2003
KJ752083 chicken South Africa 2011
PP228907 chicken South Africa 2022
OQ627800 chicken Brazil 2018
AB009628 domestic pigeon Japan 1987 G18P[17]
